# Ancient and Recent Adaptive Evolution of Primate Non-Homologous End Joining Genes

**DOI:** 10.1371/journal.pgen.1001169

**Published:** 2010-10-21

**Authors:** Ann Demogines, Alysia M. East, Ji-Hoon Lee, Sharon R. Grossman, Pardis C. Sabeti, Tanya T. Paull, Sara L. Sawyer

**Affiliations:** 1 Section of Molecular Genetics and Microbiology, and Institute for Cellular and Molecular Biology, The University of Texas at Austin, Austin, Texas, United States of America; 2 The Howard Hughes Medical Institute, Chevy Chase, Maryland, United States of America; 3 FAS Center for Systems Biology, Department of Organismic and Evolutionary Biology, Harvard University, Cambridge, Massachusetts, United States of America; University of Aarhus, Denmark

## Abstract

In human cells, DNA double-strand breaks are repaired primarily by the non-homologous end joining (NHEJ) pathway. Given their critical nature, we expected NHEJ proteins to be evolutionarily conserved, with relatively little sequence change over time. Here, we report that while critical domains of these proteins are conserved as expected, the sequence of NHEJ proteins has also been shaped by recurrent positive selection, leading to rapid sequence evolution in other protein domains. In order to characterize the molecular evolution of the human NHEJ pathway, we generated large simian primate sequence datasets for NHEJ genes. Codon-based models of gene evolution yielded statistical support for the recurrent positive selection of five NHEJ genes during primate evolution: *XRCC4*, *NBS1*, *Artemis*, *POLλ*, and *CtIP*. Analysis of human polymorphism data using the composite of multiple signals (CMS) test revealed that *XRCC4* has also been subjected to positive selection in modern humans. Crystal structures are available for XRCC4, Nbs1, and Polλ; and residues under positive selection fall exclusively on the surfaces of these proteins. Despite the positive selection of such residues, biochemical experiments with variants of one positively selected site in Nbs1 confirm that functions necessary for DNA repair and checkpoint signaling have been conserved. However, many viruses interact with the proteins of the NHEJ pathway as part of their infectious lifecycle. We propose that an ongoing evolutionary arms race between viruses and NHEJ genes may be driving the surprisingly rapid evolution of these critical genes.

## Author Summary

Because all cells experience DNA damage, they must also have mechanisms for repairing DNA. When the proteins that repair DNA malfunction, mutation and disease often result. Based on their fundamental importance, DNA repair proteins would be expected to be well preserved over evolutionary time in order to ensure optimal DNA repair function. However, a previous genome-wide study of molecular evolution in *Saccharomyces* yeast identified the non-homologous end joining (NHEJ) DNA repair pathway as one of the two most rapidly evolving pathways in the yeast genome. In order to analyze the evolution of this pathway in humans, we have generated large evolutionary sequence sets of NHEJ genes from our primate relatives. Similar to the scenario in yeast, several genes in this pathway are evolving rapidly in primate genomes and in modern human populations. Thus, complex and seemingly opposite selective forces are shaping the evolution of these important DNA repair genes. The finding that NHEJ genes are rapidly evolving in species groups as diverse as yeasts and primates indicates a systematic perturbation of the NHEJ pathway, one that is potentially important to human health.

## Introduction

DNA double-strand breaks are a particularly toxic form of DNA lesion. Such breaks are repaired through several pathways, the most well-studied being homologous recombination and non-homologous end joining (NHEJ; reviewed in [1]). NHEJ is also required for V(D)J recombination, which generates immunoglobulin and T cell receptor diversity. Accordingly, mutations in NHEJ genes have been linked to both cancer and immune deficiencies. Given the central importance of these processes, NHEJ genes are expected to have a low tolerance for mutations. Such a hypothesis would be supported if sequences of NHEJ genes are stable and relatively unchanging over evolutionary time.

In contrast to this expectation, a genome-wide analysis uncovered NHEJ as one of the two functional pathways most enriched for positive selection during *Saccharomyces* evolution [2]. Positive selection occurs when natural selection operates on an advantageous mutation, driving an increase in its prevalence over time, and sometimes leading to fixation of this mutation in the species in which it arose. Because advantageous mutations commonly involve a change in protein sequence, recurrent rounds of positive selection can lead to relatively rapid protein sequence evolution over time. Positive selection has been found to predominantly affect genes in three functional classes: reproduction, immunity, and environmental perception (smell, taste, etc), presumably because these processes are under strong selection for constant adaptive change [3]–[10]. The intriguing observation of positive selection in the NHEJ genes of *Saccharomyces* remains unexplained, but could potentially be attributed to the fact that NHEJ is not the major pathway for the repair of double-strand breaks in yeast [11]. Relaxation of evolutionary constraints on NHEJ genes in yeast species, due to their reliance predominantly on the homologous recombination pathway, could have made NHEJ genes vulnerable to competing evolutionary forces. In this study, we have analyzed the molecular evolution of NHEJ genes in primates, including humans, where NHEJ is the major pathway for DNA double-strand break repair.

NHEJ is activated upon detection of DNA double-strand breaks. After detection, NHEJ proteins enzymatically process broken DNA ends to allow for efficient end joining. Repair is then completed through the action of repair-specific DNA polymerases and the NHEJ ligation complex, which fill in and seal the break [1]. To analyze the selective pressures that have shaped the genes of the human NHEJ pathway, we generated sequence datasets of primate orthologs from twenty simian primate species. We find support for positive selection in five NHEJ genes: *NBS1*, *CtIP*, *Artemis*, *XRCC4* and *POLλ*. Analysis of human polymorphism data indicates that positive selection has also operated on *XRCC4* in modern humans. Crystal structures are available for the Nbs1, XRCC4, and Polλ proteins, and in all cases we find that amino acid sites targeted by positive selection fall on protein surfaces. It is well-established that rapidly evolving amino acid residues tend to be found on the surfaces of proteins [12]–[14]. In previous studies where the significance of these residues has been structurally or functionally investigated, it has been shown that they modulate protein-protein, protein-ligand, or protein-DNA interactions [15]–[24]. However, we demonstrate biochemically that positive selection in Nbs1 at one of the three residues identified has not affected its physical interactions with other DNA repair components. In the discussion, we propose that the positive selection of NHEJ genes may be explained by the diverse viruses and genetic parasites that interact with these proteins to promote their own lifecycle.

## Results

### Sliding window analysis of selective pressures shaping NHEJ genes

**Figure 1:**
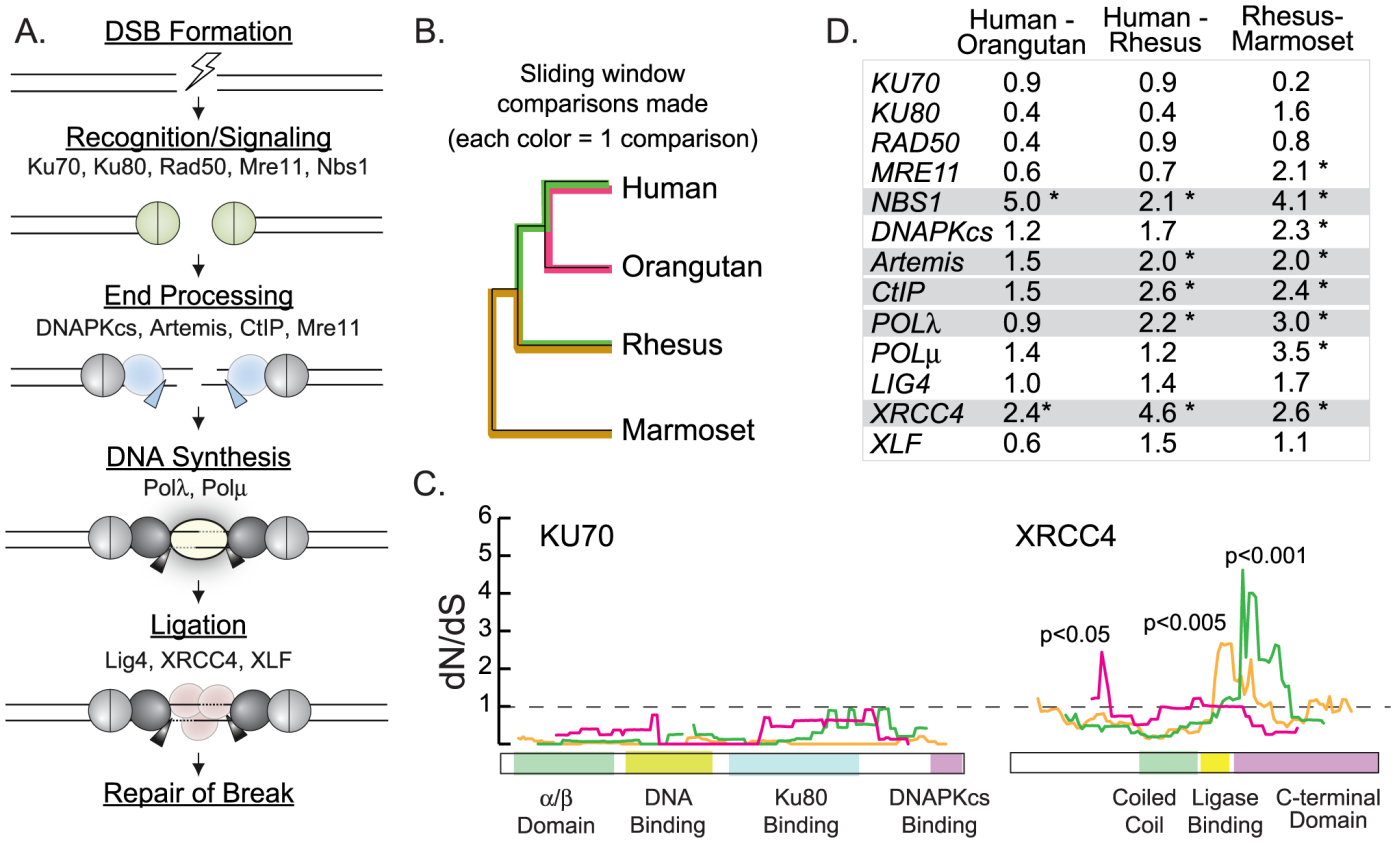
Sliding window analysis identifies five candidate NHEJ genes evolving under positive selection. A) A schematic of the mammalian non-homologous end joining pathway is shown, illustrating the roles of all proteins included in this study. B) A cladogram shows the relationship of the primate species used in the sliding window analysis. Branch colors correspond to the sliding window comparisons graphed in panel C. C) Sliding window analysis of dN/dS along the length of *KU70* and *XRCC4*. In each case, three pairwise sequence alignments were analyzed (human - orangutan comparison in pink, human - rhesus comparison in green, and rhesus - marmoset comparison in orange). D) The table summarizes the maximum dN/dS peak height found along the length of each pairwise sliding window comparison made. Asterisks indicate statistically significant peaks (p<0.05). In gray highlight are the five genes with significant peaks in at least two out of three comparisons.

We utilized primate sequence datasets to study the evolutionary history of human NHEJ genes. With human population genetic data, evolutionary pressures can usually only be summarized for chromosomal regions larger than a single gene. However, with inter-species divergence data, resolution of evolutionary signatures can be increased to the level of a single gene, and it is sometimes possible to see the serial fixation of mutations in particular gene regions or even codons. The limitation in these studies is the number of available primate sequences. We first performed a preliminary survey of the selective pressures that have shaped all of the major genes of the NHEJ pathway (Figure 1A), so that we could generate appropriate primate datasets for candidate genes containing signatures suggestive of positive selection.

Five nearly complete primate genome projects are publicly available: human, chimpanzee, orangutan, rhesus macaque, and marmoset. Ten possible pairwise gene comparisons can be made between these five species, but three pairwise comparisons (human-orangutan, human-rhesus, and rhesus-marmoset) were chosen that maximize divergence and minimize phylogenetic re-sampling (Figure 1B). For each NHEJ gene, these three pairwise gene alignments were constructed and analyzed with a custom algorithm that calculates dN/dS in sliding windows along the length of each gene [25]. The dN/dS ratio captures the ratio of non-synonymous (dN; changing the encoded amino acid) to synonymous (dS; silent) DNA mutations that have accumulated since two genes last shared a common ancestor [26]. For most protein-encoding genes, the observed number of non-synonymous mutations is far less than the number of synonymous mutations observed (dN/dS<1) [5]. This is because mutations which cause an alteration in amino acid sequence are more likely to be detrimental to proper protein folding and function, and are therefore typically selected against (purifying selection). As expected in a typical gene, dN is less than dS (dN/dS<1) for all windows along the length of the NHEJ gene *KU70* (Figure 1C).

Under positive selection, non-synonymous mutations are swept through populations more quickly than neutral or nearly-neutral synonymous mutations due to a selectable advantage that they convey. After many such rounds, such a regime gives rise to the dN>dS signature that is indicative of positive selection (dN/dS>1). Sliding window analysis of dN/dS is useful when making pairwise gene comparisons, as positive selection may be limited to specific regions that are buried within a gene that is otherwise conserved. In the case of *XRCC4*, sliding window analyses of human-rhesus and rhesus-marmoset pairwise alignments highlight the 3′ end of the gene as having signatures of dN/dS>1 (p<0.001 and p<0.005, respectively; Figure 1C). In this region, human and rhesus *XRCC4* sequences differ by nine non-synonymous DNA mutations and zero synonymous mutations. In the human–orangutan comparison, a different region in the 5′ end of the gene shows a significant inflation of dN/dS above 1 (p<0.05). The different location of this signal may indicate a unique selective force that is operating specifically in the great apes.

Sliding window analyses have an inherent multiple testing problem that is difficult to correct because of the non-independence of tests (windows overlap) [27]. Nevertheless, we have successfully utilized sliding window analysis as a pre-screening tool in several previous studies [2], [28]. As an ad hoc method for eliminating some false positive signatures, we sought genes with regions of dN/dS significantly>1 in at least two out of three different pairwise primate comparisons made. All pairwise comparisons for each NHEJ gene are shown in Figure S1, and the maximum dN/dS value found in each comparison is summarized in Figure 1D. We find that five out of thirteen NHEJ genes bear significant regions of dN/dS>1 in at least two out of the three primate comparisons made (highlighted in gray in Figure 1D). Thus, we have identified preliminary signals of positive selection in five candidate NHEJ genes: *NBS1*, *Artemis*, *CtIP*, *POLλ*, and *XRCC4*.

### Analysis of extended primate datasets for candidate genes

In order to verify positive selection with greater statistical rigor, larger sequence datasets are required. We sequenced all five candidate genes from 15 additional hominoid, old world monkey, and new world monkey species. Despite the fact that no significant windows of dN/dS>1 were observed in any of the pairwise comparisons of *XLF* (Figure 1D), we also included this gene because positive selection was previously reported in an analysis of mammalian *XLF* sequences [29]. In total, 90 primate genes were sequenced (6 genes, each from 15 species). We also re-sequenced all genes that were incomplete in the available primate genome projects (chimpanzee, orangutan, rhesus macaque, or marmoset). Details of primate cell lines, cell culture, mRNA extraction, cDNA library construction, and divergent-species PCR are given in the materials and methods section and in Tables S1, S2, S3. The resulting dataset for each gene is comprised of orthologs from 20 primate species that represent approximately 35 million years of primate evolution [30].

The multiple sequence alignment generated for each gene was analyzed for positive selection with the “codeml” program in PAML [31]. The codeml program provides a maximum likelihood framework for estimating dN/dS rates over the entire history of primate evolution by integrating over all ancestral gene sequences in the context of a phylogeny [32], [33]. This program offers several models for gene evolution, some where no codons are allowed to evolve with dN/dS>1 (NSsites models M1a, M7 and M8a), and others where positive selection of some codons is allowed (NSsites models M2a and M8). A likelihood ratio test allows comparison of positive selection models to null models. Results of all model comparisons for each gene are provided in Tables S4, S5, S6, S7, S8, S9, and the results of the M8a vs. M8 comparisons, using the f61 model of codon usage, are summarized in Table 1. The null model (M8a) is rejected (p<0.05) in favor of the model of positive selection (M8) in four of these six genes: *CtIP*, *Artemis*, *XRCC4*, and *POLλ*. For *NBS1*, the null model was very nearly rejected (p = 0.056). This analysis did not support a model of positive selection in primate *XLF* (p = 0.59). As mentioned above, sliding window analysis did not detect domains of positive selection in *XLF*. In conclusion, we find strong support for positive selection in four genes of the primate NHEJ pathway, a surprising finding given the critical role that these proteins play in DNA repair.

**Table 1:** PAML analysis of primate NHEJ genes.

Gene^a^	2Δl^b^	p-value^b^	dN/dS^c^	% sites^c^	Codons with dN/dS>1^d^
*NBS1*	3.7	p = 0.056	5.3	1.1%	G9
*NBS1* hominoids only	3.9	p = 0.048	7.6	2.3%	G9 , E185 , I531**
*CtIP*	8.4	p<0.004	2.1	14.8%	C155, I187*, M235, T333*, I336, K355**, S365*, C368, I399, L416*, N420*, G425**, M481, V486*, K515*, G541**, T544, C554, S574*, S605*, D619, L724, R730
*Artemis*	5.5	p<0.02	2.1	11%	I83*, N250**, T365*, F411*, M418**, E439*, V463*, G484*, S503*, T511, A576**, K610*, S626*
*XRCC4*	8.5	p<0.004	15	0.6%	L243**
*XRCC4* head domain (aa 1–115)	0.06	p = 0.81	not sig.	not sig.	-
*XRCC4* CC domain (aa 116–203)	<0.01	p>0.99	not sig.	not sig.	-
*XRCC4* C-term domain (aa 204–336)	21	p<0.001	8.7	8.1%	R205**, Q211*, A216*, C218**, L243**, Q292
*POLλ*	12	p<0.001	3.2	5.4%	Q102*, S167*, A208*, P231*, E330, S381*, R441*, R484**
*XLF*	0.29	p = 0.59	not sig.	not sig.	-

a Each analysis was performed on a dataset consisting of 20 primate sequences (for species list see Table S1), with the exception of the *Artemis* (due to deletions/missing sequence in two species) and *NBS1* “hominoids only” analyses. See Table S6 (*Artemis*) and Table S4 (*NBS1*) for lists of primates used in these analyses.

b Twice the difference in the natural logs of the likelihoods (2Δl) of the two models (M8a-M8) being compared. The p-value indicates the confidence with which the null model (M8a) can be rejected in favor of the model of positive selection (M8).

c dN/dS value of the class of codons evolving under positive selection in M8, and the percent of codons falling in that class.

d Codons assigned to the class evolving under positive selection in M8 with a posterior probability >0.90 by naive empirical bayes (NEB) analysis (* p>0.95, ** p>0.99). Coordinates correspond to the human protein.

Analysis of the 20-species *NBS1* dataset yielded marginal support for positive selection (p = 0.056; Table 1). However, we noticed that several amino acid positions in the NBS1 protein alignment had changed multiple times exclusively in hominoid species (humans, great apes, and gibbons). Based on this, we considered that positive selection of *NBS1* may be specific to hominoids. Indeed, analysis of *NBS1* from only the hominoid species resulted in improved statistical support for positive selection (p = 0.048; Table 1), despite the fact that the analysis of only eight sequences should greatly reduce statistical power. To formally test the hypothesis of hominoid-specific positive selection, we analyzed our datasets with “branch-site” models of evolution [34]. This test allowed us to determine whether there are codon positions evolving under positive selection specifically in the hominoid clade. *NBS1* was the only one of the six NHEJ genes for which this hypothesis was supported (p<0.005; Table S10), and support is robust under all models of codon usage (Table S11). Because three total tests were performed on the *NBS1* dataset, a Bonferroni-corrected p-value can be calculated for the rejection of the null hypothesis in the branch-sites test (p<0.015). Thus, hominoid-specific positive selection is supported in *NBS1*. Interestingly, the yeast ortholog of *NBS1 (XRS2)* was also identified as being under positive selection during *Saccharomyces* evolution [2].

**Figure 2:**
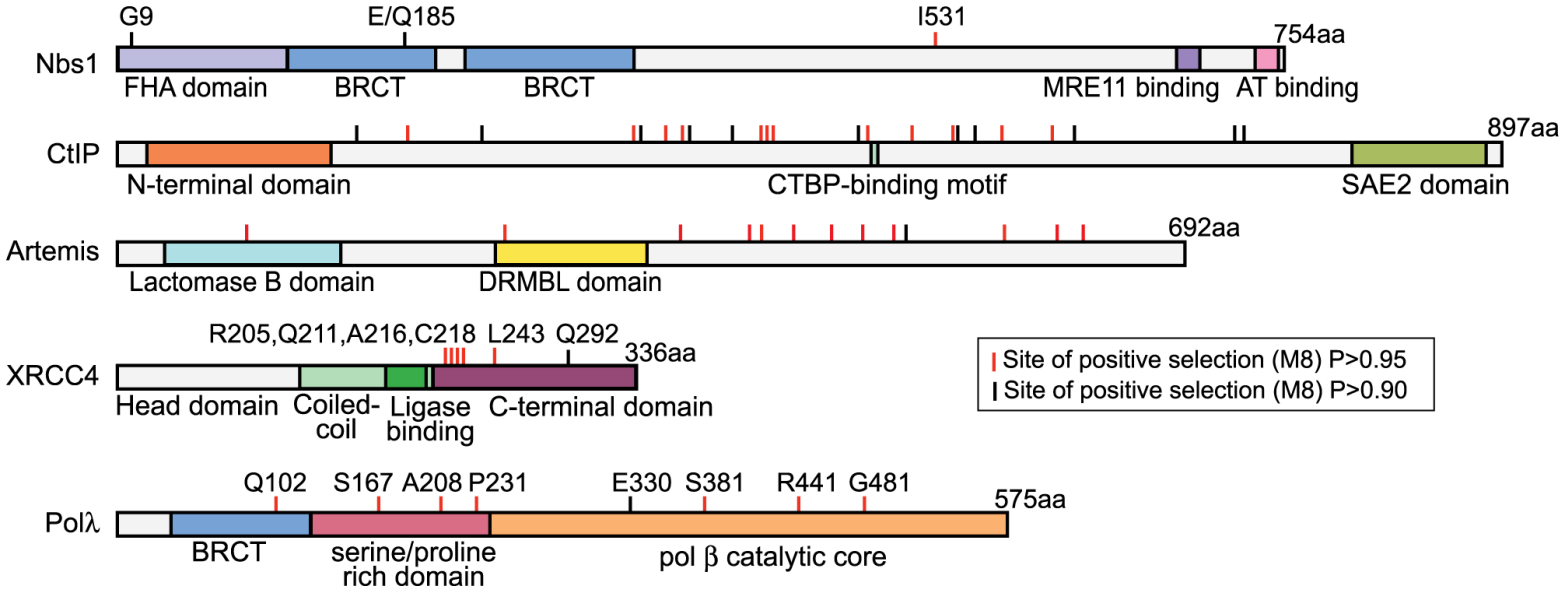
Five proteins in the NHEJ pathway show signatures of positive selection. Domain diagrams are shown for the five NHEJ proteins evolving under recurrent positive selection during primate speciation. The locations of specific amino acid positions under positive selection have been marked on these diagrams (red tick mark indicates posterior probability of >0.95, black tick mark indicates posterior probability of >0.90). Amino acid positions specifically discussed in the text are indicated.

Specific codon sites that have been the target of recurrent positive selection could be identified in the dataset for each NHEJ gene (Table 1). Posterior probabilities of codons included in the dN/dS>1 site class are commonly considered highly significant at cutoffs as low as P = 0.90, and potentially even lower [35]. The positions of these amino acid sites are summarized in Figure 2. Crystal structures have been solved for Polλ, XRCC4, and Nbs1, allowing us to further analyze the patterns of positive selection in these three proteins.

### Positive selection of *POLλ*

**Figure 3:**
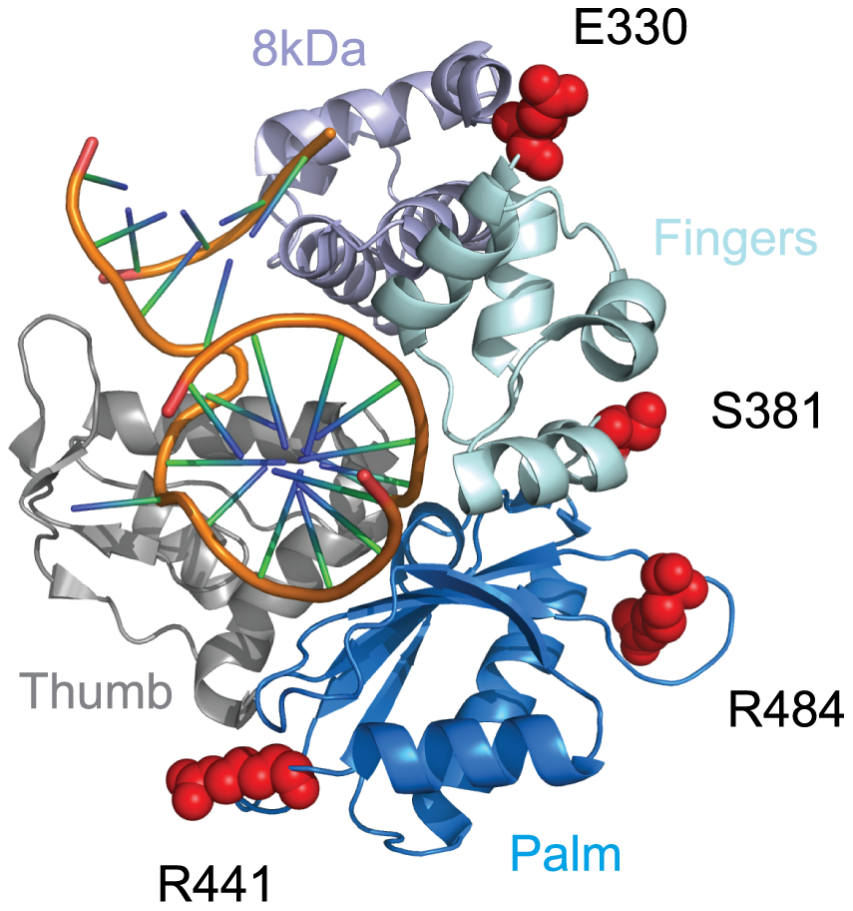
Residues under positive selection in Polλ fall on the protein surface. The co-crystal of the human Polλ 39 kDa catalytic domain in complex with a DNA substrate is shown (PDB 2BCQ) [37]. Four of the eight amino acid positions found to be under positive selection (330, 381, 441, and 484; red globes) could be mapped onto the structure. All four fall onto the outer surface of the protein and are not predicted to interfere with the polymerase active site.

Polλ is one of two DNA polymerases involved in the filling of gaps formed during NHEJ [36]. Approximately 5% of the codons in this gene were identified as evolving under positive selection, with an average dN/dS value of 3.2 (Table 1). Eight specific codons could be assigned to this class with high posterior probability (P>0.90), and these sites are scattered across the linear protein sequence (Figure 2). The crystal structure of the 39 kDa Polλ catalytic domain has been solved in complex with substrate DNA, and this catalytic core is comprised of the fingers, palm, thumb, and 8 kDa subdomains (Figure 3) [37]. Four of the eight amino acid sites identified as being positively selected are part of this catalytic core domain. All four (E330, S381, R441, and R484) map to the outer surface of the three-dimensional structure (red balls in Figure 3), with none of the sites being found within the enzyme active site. Thus, residues under recurrent positive selection fall on the protein surface, and mutations at these sites are not predicted to directly affect catalytic activity.

### Positive selection of *XRCC4* during primate evolution

The NHEJ-specific ligase complex is composed of DNA ligase IV (Lig4) along with the regulatory molecules XLF and XRCC4 [1]. The dN/dS>1 site class in *XRCC4* is assigned a value of dN/dS = 15, nearly double the value seen for any other NHEJ gene (Table 1). Given the extreme value, only one codon, L243, can be supported as a member of this class with high posterior probability (P>0.99). To uncover more codons that may be evolving under positive selection, a secondary analysis was performed on the three XRCC4 structural domains: the N-terminal head domain, which is involved in DNA binding, the coiled-coil stalk domain, which includes the ligase binding domain, and the unstructured C-terminal domain (residues 204–336). Positive selection is supported only in the C-terminal domain (p<0.001; Table 1). Because four tests were performed on the *XRCC4* dataset, the Bonferroni-corrected p-value for the observation of positive selection in the C-terminal domain is p<0.004. In this domain, six codon sites, including L243 identified previously, were identified as evolving under positive selection (P>0.90), with support for five of these being P>0.95. These codons were now collectively assigned a dN/dS value of 8.7. All of these codons were also identified, albeit with lower confidence, in the full-length *XRCC4* analysis (Table S7).

**Figure 4:**
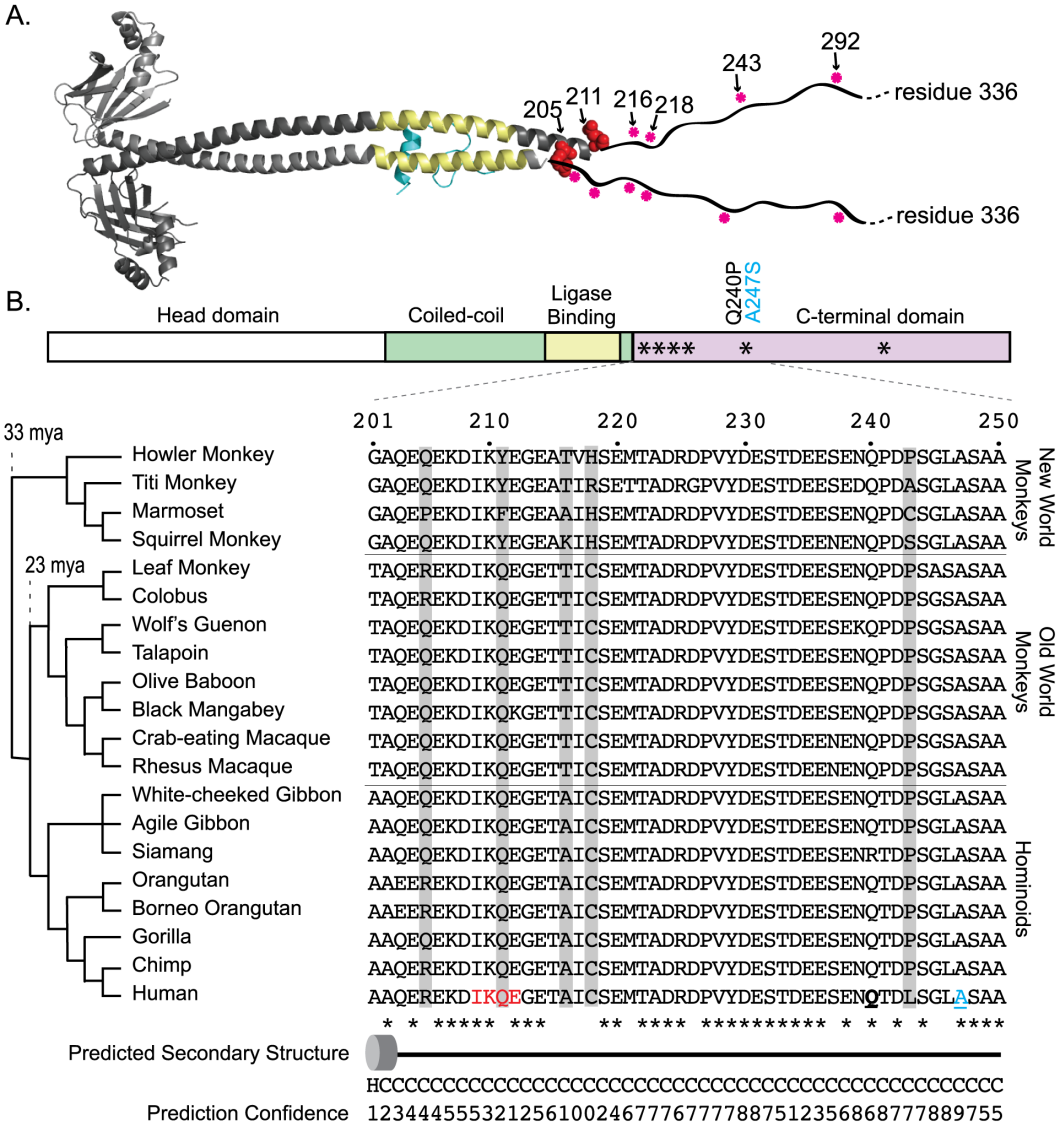
XRCC4, a component of the NHEJ ligase complex, shows a clustered signature of positive selection. A) A co-crystal of the human XRCC4 homodimer (grey) in complex with a fragment of its binding partner Lig4 (blue) has been solved (PDB 1IK9) [38]. The ligase-binding domain of XRCC4 is shown in yellow. The C-terminal domain of the 336 amino acid protein is unstructured and had to be truncated for crystallization. This portion has been artificially indicated by the wavy black line. In the crystal structure, the two monomeric chains are different lengths. Chain A (dark gray) is comprised of residues 1–211, while chain B (light gray) is comprised of residues 1–201. Two of the amino acids positions found to be under positive selection (205 and 211; red globes) could be mapped only to the longer of the two monomers (chain A). Sites 216, 218, 243, and 292 could not be mapped to either monomer. Their approximate location is marked with a pink asterisks on the linear schematic of the C-terminal domain. B) A linear domain diagram of XRCC4 is shown, with the approximate location of the amino acid sites under positive selection marked with asterisks. An amino acid alignment in this region for the 20 primate species used in this study is shown, with residues found to be under positive selection highlighted in gray. Residue 211, which was identified as being subject to positive selection, lies at the third position within the SUMOylation consensus site (IKQE; denoted in red), with the neighboring lysine being SUMOylated [41]. Another amino acid position that has evolved under positive selection, residue 243, is located just four positions upstream of a A247S human disease mutation which has been linked to oral cancer susceptibility [43], and three positions downstream of the human Q240P polymorphism (these two sites are underlined in the human amino acid sequence). Secondary structure predictions and confidence values (0, low; 9,high) were obtained with the PSIPRED server [93]. “H” and the barrel shape denote the very end of the long alpha helix that is observed in the crystal structure. Downstream of this, “C” indicates the unstructured region.

The partial crystal structure of the XRCC4 dimer in complex with its binding partner, Lig4, has been solved [38]. All six of the identified codons map just downstream of the Lig4-binding domain (red dots in Figure 4A), in a region of the protein where the structure is predicted to transition from an alpha-helix to an unstructured domain. This unstructured domain is not included in the crystal structure, but has been represented in schematic form for illustration. Strikingly, of the five sites supported at the 95% confidence level, the first four (R205, Q211, A216, and C218) lie within a 14 amino acid stretch of the protein (4% of the length of the protein), and the fifth site (L243) lies just 25 residues downstream of this cluster. We assessed the significance of this clustering on the linear protein sequence by determining how many times a random sampling of five sites fell in a cluster equal to or smaller than the 39 amino acid region that contains the sites under positive selection. Comparing this observed distance to a null distribution (100,000 permutations) lends statistical support to the hypothesis that these positively selected sites are clustered (p = 0.0005). The functional significance of this “patch” of positive selection is unknown. A protein alignment of primate XRCC4 in this region is shown in Figure 4B. To the left, a cladogram shows the relationship of the twenty primate species used in this study. Amino acid positions evolving under positive selection are shown in the alignment in gray. This unstructured C-terminal domain has been shown to be dispensable for repair and V(D)J recombination [39], [40]. However, this domain also contains a number of regulatory sites including a SUMOylation site and several DNA-PKcs phosphorylation sites [41], [42], as well as a known cancer-linked mutation [43] (Figure 4B).

### Positive selection of *XRCC4* in modern humans

**Figure 5:**
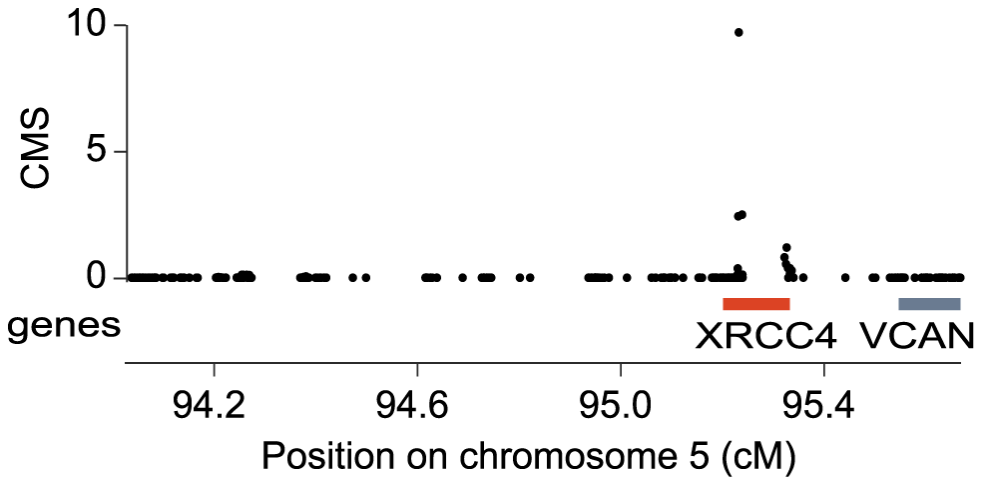
*XRCC4* is under positive selection in modern humans. CMS analysis of *XRCC4* in the CEU population. The CEU population represents humans with ancestry from northern and western Europe. Bars on the x-axis indicate genes (red bar indicates *XRCC4*; grey bar indicates *VCAN*), and black dots show CMS values.

We investigated whether the NHEJ genes that have been subject to ancient recurrent positive selection in simian primates are also under recent local adaptation in humans. We examined the five genes *POLλ*, *XRCC4*, *Artemis*, *NBS1*, and *CtIP* for signals of selection in the HapMap Phase II [44] data using a recently published method, the Composite of Multiple Signals (CMS) [45]. By combining multiple tests, CMS increases resolution for localizing signals of selection by up to 100-fold, and has a lower false-positive rate than the component individual tests. We examined SNPs within and surrounding each gene of interest, with a window size of 100kb upstream and 100kb downstream of each gene (see Materials and Methods). In the European population, the CMS signal for *XRCC4* is significant at a threshold that yields a 0.1% false positive rate in simulations, and is one of the top 60 strongest signals in the genome (Table S12). Applying CMS to fine-map the region, we localized the signal to 83kb entirely within the gene, suggesting that *XRCC4* is a target of recent local adaptation (Figure 5). In the other four genes, we did not observe any signals significant at the same level as *XRCC4*, but we do observe suggestive signals by the individual tests (in the top 1–5% tail genome-wide) in *POLλ* and *XRCC4* in the West African population, and *Artemis* in the European population (Table S12). As CMS is optimized to detect recent local adaptation in a single population, these signals by individual tests may reflect selective events outside of this model (e.g., selection on standing variation, or selection of the same allele in multiple populations). Indeed, a single allele of *POLλ* has previously been reported to be under positive selection in both Asian and Sub-Sahara African populations [46]. Thus we find that several of the genes that have been evolving under positive selection during primate evolution also show evidence suggestive of recent positive selection in human populations, with an especially strong signature identified in *XRCC4*.

### Essential repair-related interactions are conserved despite positive selection of Nbs1

**Figure 6:**
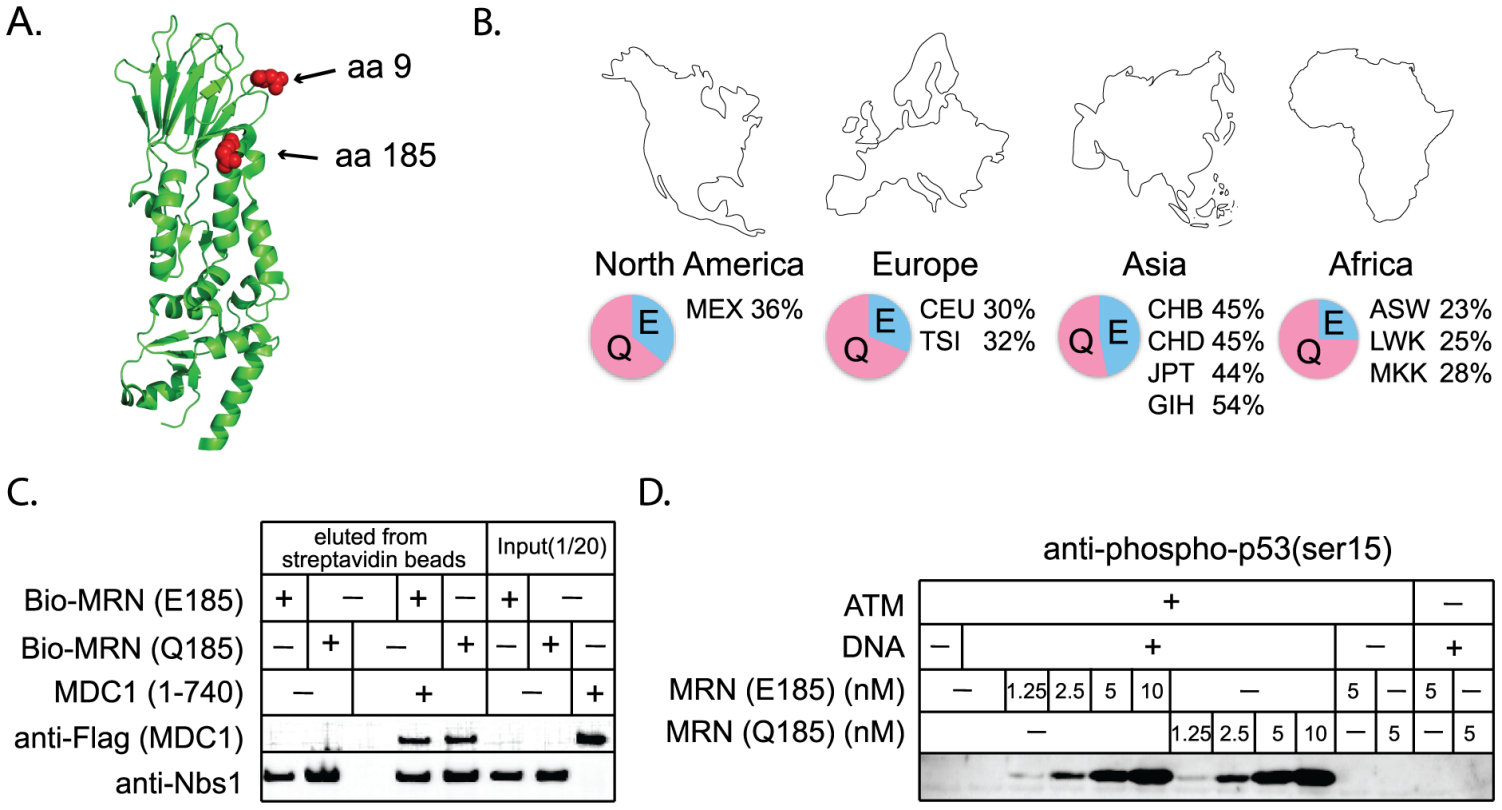
Interactions with other repair proteins have been conserved in Nbs1 despite its positive selection. A) Positively selected residues 9 and 185 (red balls) are mapped onto the partial Nbs1 structure (PDB 3HUE) [48]. B) SNP frequencies of Q185E are reported for the ten human populations included in the HapMap project (http://hapmap.ncbi.nlm.nih.gov/). Three-letter labels are standard codes (ASW - African ancestry in Southwest USA; CEU - Utah residents with Northern and Western European ancestry; CHB- Han Chinese in Beijing, China; CHD - Chinese in Metropolitan Denver, Colorado; GIH - Gujarati Indians in Houston, Texas; JPT - Japanese in Tokyo, Japan; LWK - Luhya in Webuye, Kenya; MEX - Mexican ancestry in Los Angeles, California; MKK - Maasai in Kinyawa, Kenya; TSI - Toscans in Italy). C) Binding assays were performed between recombinant biotinylated MRN complexes containing Nbs1 E185 or Q185, and an N-terminal Flag-tagged fragment of Mdc1 containing amino acids 1 to 740, as indicated. The biotinylated MRN complexes (20nM) were incubated with 45 nM Mdc1 and then isolated with streptavidin-coated magnetic beads. Bound protein was visualized by western blotting with anti-Flag (Mdc1) and anti-Nbs1 antibodies. D) MRN complexes containing Nbs1 E185 or Q185 were tested in ATM kinase assays with linear DNA as indicated. Phosphorylation of the substrate, GST-p53 (aa 1–100), was assessed by western blotting using a phospho-specific antibody directed against p53-phospho-ser15 as previously described [57].

Nbs1 is part of the MRN complex, containing Mre11, Rad50, and Nbs1. This complex is involved in DNA break detection, end processing, and cellular signaling [47]. Mutations in *NBS1* lead to the autosomal recessive disease, Nijmegen breakage syndrome, which is characterized by chromosomal instability. Three amino acid positions were identified as evolving under positive selection (Table 1). G9, Q185, and I531 are identified with P>0.90, with support for I531 being P>0.99. A partial Nbs1 structure is available [48], and two of the amino acid sites targeted by positive selection (residues 9 and 185) fall on the protein surface (Figure 6A). The third site, residue 531, is not included in this partial structure.

The positive selection of NHEJ genes suggests that certain mutations are providing a fitness advantage in an unknown context. While the essential DNA repair functions of these genes would be expected to remain conserved, there is a formal possibility that adaptive evolution of NHEJ genes could come at the cost of DNA repair. We wished to consider this hypothesis because a human SNP at a site of positive selection in *NBS1* (Q185E; SNP ID rs1805794) has been linked to increased risk of renal, skin, and lung cancer in multiple association studies [49]–[52]. This SNP is found at high frequencies in human populations (Figure 6B). While Q185E has been linked to cancer, association studies are limited in that they may identify either a causal SNP, or a SNP that is linked to a causal SNP. We wished to test whether amino acid substitution in this codon changes the performance of Nbs1 in DNA repair, as the association with cancer might suggest.

We constructed *NBS1* alleles encoding either an E or a Q at position 185, and expressed these proteins in insect cells using a baculovirus system. We then tested the effects of this mutation on several of the known activities of Nbs1. The Nbs1 N-terminus, including the BRCT domain in which this SNP is located, is known to bind to the checkpoint protein Mdc1 [53]–[56]. We produced and purified MRN complexes containing both versions of Nbs1 and find that both interact equally well with purified Mdc1 in an *in vitro* binding assay (Figure 6C). Thus the Nbs1 E/Q polymorphism is not expected to affect the association of MRN with Mdc1 at sites of DNA damage *in vivo*. The MRN complex is also required for the activation of the checkpoint protein ATM [57], [58]. We find that MRN complexes containing both versions of Nbs1 are equally efficient in stimulating ATM-dependent phosphorylation of one of the downstream targets of ATM, p53 (Figure 6D). Nbs1 is also known to bind XRCC4/Lig4 [59] and we find that both versions of Nbs1 interact equally well with this complex *in vitro* (data not shown). Therefore, we conclude that positive selection of this codon, regardless of what is driving it, has not affected the repair-related physical interactions of Nbs1. However, it should be noted that laboratory-based assays may not be sensitive enough to detect subtle defects that could cause a minor fitness effect in nature.

## Discussion

The NHEJ pathway is over 3 billion years old, and is found in bacteria, archaea, and eukaryotes. Despite the ancient conservation of the pathway, we have identified five NHEJ genes that have evolved under positive selection during the evolution of simian primates: *NBS1*, *CtIP*, *Artemis*, *XRCC4*, and *POLλ*. An analysis of polymorphism data supports positive selection of *XRCC4* in modern humans as well. Interestingly, the yeast ortholog of *NBS1 (XRS2)* was also identified as one of the two *Saccharomyces* NHEJ genes with the most extreme signatures of positive selection [2]. One hypothesis is that these signatures of positive selection are reflective of natural selection for more efficient DNA repair. As certain NHEJ components evolve, compensatory mutations may arise in other NHEJ components to re-optimize protein-protein interactions between the various components. We feel that this model is unlikely. In the absence of an antagonizing force, there is no reason that recurrent adaptive change should be required of any member of this pathway, which would then need to be followed by compensatory change. Four observations from our study additionally argue against this model. First, our biochemical experiments with Nbs1 suggest that positive selection of at least one of the three sites identified has not altered interactions with other repair proteins. Second, although there are several core complexes involved in NHEJ (the MRN complex and the Lig4/XRCC4/XLF complex), only one component of each of these was identified as evolving under positive selection. Third, the clustered sites of positive selection in XRCC4 fall within the C-terminal protein domain that is not essential for DNA repair. Fourth, the positive selection of the NHEJ pathway is not a primate specific phenomenon, but is also found in *Saccharomyces* yeast [2], arguing against a model where some novel role for DNA repair during primate evolution has driven this selection.

The finding of multiple primate NHEJ components evolving under positive selection, supported by parallel findings in *Saccharomyces* yeast, indicates a systematic perturbation of the NHEJ pathway. With positive selection observed in two highly divergent eukaryotic clades, a model for the cause of this rapid evolution must span such diverse species groups. We propose that NHEJ genes may be antagonized by genetic parasites, which in primates are comprised of viruses and retrotransposons.

Proteins of the NHEJ repair pathway have been shown to act as antiviral factors in the lifecycle of human adenovirus, a linear double-stranded DNA virus. Adenoviruses are a major cause of upper respiratory and other infections in humans. During infection, components of the NHEJ pathway join together viral genome ends, causing “dead-end” viral genome concatenation [60]. To counteract this antiviral tactic, adenovirus proteins (encoded by the E4 genes) sequester and target for degradation a number of components of the NHEJ pathway, including components of the Mre11/Rad50/Nbs1 and Lig4/XRCC4/XLF complexes [60]–[63]. CtIP has also been implicated in the adenovirus lifecycle through its interaction with the adenovirus early region 1A (AdE1A) protein [64]. If primate NHEJ genes are continually selected to encode variants that can evade interaction with these adenoviral antagonists, while the viral antagonists continually counter-evolve, this could drive positive selection of primate NHEJ genes. Adenovirus has been found in stool samples from great apes and macaques [65], indicating a possible long-standing co-evolution between this virus and primates.

Retroviruses like HIV may also provide the selective pressure that shapes the recurrent positive selection of NHEJ genes. There is abundant genetic evidence suggesting a role for NHEJ in the retroviral lifecycle [66]–[70]. Upon cellular entry, the retroviral RNA genome is reverse transcribed into double-stranded DNA. The ultimate destination for this retroviral cDNA is integration into the genome of the host, but it must first survive passage through the nucleus without being detected as broken DNA by the cell. NHEJ proteins have been found to physically associate with retroviral proteins, cDNA, and pre-integration complexes *in vivo* and in two-hybrid interactions [67], [71]–[74]. There are several models which have been proposed to explain this. In one model, NHEJ proteins are recruited by the viral complex to protect free viral cDNA ends from degradation or from triggering apoptosis. In another model, the viral complex recruits host NHEJ proteins to promote the repair of breaks created at sites of retroviral cDNA integration into the host genome. In a third model, NHEJ proteins act as antivirals, joining the two long-terminal repeat (LTR) ends of the viral cDNA into dead-end “2-LTR circles.” These 2-LTR circles are ubiquitously observed in the nuclei of infected cells [67]. Regardless of the model, allelic variants of NHEJ genes that result in lower infection rates would be selectively advantageous to the host. Should such alleles go to high frequency or fixation, retroviruses would be expected to counter-evolve, and the back-and-forth interplay would drive recurrent positive selection of NHEJ genes. Retroviruses and primates have co-evolved for tens of millions of years, as illustrated by the fact that all sequenced primate genomes contain the remnants of hundreds of thousands of integrated retroviruses [75].

It is unknown whether the positive selection observed in NHEJ genes represents a response to a single selective force, or whether multiple forces are shaping their evolution. At least eight additional viral families have been shown to evade or exploit the host DNA damage response [76]. Several NHEJ proteins include one or more “BRCT” domains, which have been linked to viral infection in multiple instances. The Epstein-Barr viral protein Zta has been shown to interact with the BRCT domains of 53BP1, a component of the DNA damage response, to prevent apoptosis that is activated in response to viral replication [77]. HIV-1 Tat has also been shown to interact with the BRCT domain of the human replication protein FCP1 [78]. In both Polλ and Nbs1, we find an amino acid position at the C-terminal end of the BRCT domain to be evolving under positive selection (Q185 in Nbs1 and Q102 in Polλ). The single site found to be under positive selection in *Saccharomyces* Xrs2 also falls near the end of the BRCT domain (site 298) [2]. BRCT domains could be a critical link in the interaction between viruses and the NHEJ pathway. Antagonism of host NHEJ proteins by genetic parasites may be a universal feature of cellular life, as yeast Ty retrotransposons also interact genetically and physically with NHEJ machinery [79], [80]. LINE-1 retrotransposons are major drivers of primate genome evolution, and LINE-1 retrotransposition rates are reduced in the absence of NHEJ genes [81]. The Corndog and Omega bacteriophages of mycobacteria have even incorporated the first gene in the bacterial NHEJ pathway, Ku, into their own genome [82]. This viral Ku now evolves under the selective pressures of the virus in order to recruit the bacterial NHEJ ligase, LigD, to circularize phage DNA.

In summary, we have documented abundant signatures of positive selection in genes of the NHEJ pathway, which is the major pathway for repairing double-strand chromosomal breaks in mammalian cells. We propose the hypothesis that these signatures result from the long-term co-evolution between NHEJ genes and genetic parasites. While it is well known that genetic parasites shape genome architecture through insertion and subsequent inter-element recombination, the present study may indicate that selective pressures imposed by genetic parasites can drive the evolution of protein sequence in critical human proteins.

## Materials and Methods

### Primate NHEJ gene sequences

Chimpanzee, orangutan, rhesus macaque, and marmoset gene sequences were obtained from the UCSC genome database (http://genome.ucsc.edu/) using the BLAT alignment tool [83]. *NBS1*, *CtIP*, *Artemis*, *XRCC4*, *POLλ*, and *XLF* were sequenced from 15 additional primate species, and poor-quality regions of chimpanzee, orangutan, rhesus and marmoset genes were also re-sequenced. Primary and immortalized primate cell lines (sources and individual primate identifiers are listed in Table S1) were grown in standard media supplemented with 15% fetal bovine serum at 37°C and in 5% CO_2_. Total RNA was harvested from cell lines using the AllPrep DNA/RNA kit (Qiagen). PCR was performed from total RNA and/or cDNA with OneStep RT-PCR kit (Qiagen) or PCR SuperMix High Fidelity (Invitrogen), respectively. Details of the PCR and sequencing strategy, along with primer sequences, can be found in Tables S2 and S3. Primate NHEJ gene sequences have been deposited in GenBank (accession numbers HM486750–HM486849).

### Sliding window analysis

Alignments between orthologous gene pairs were performed using ClustalX2.0 [84]. Sliding-window dN/dS calculations for each alignment were performed with the SLIDERKK program [25]. Human-orangutan, human-rhesus and rhesus-marmoset alignments were analyzed with standard window sizes of 450bp, 306bp and 153bp, respectively, to reflect the increasing level of divergence in these species pairs (window size must be a multiple of nine in this program) [2], [28]. In order to generate confidence values for windows with dN/dS>1, the K-estimator program [85] was utilized to generate a null distribution through Monte Carlo simulation of randomly derived dN/dS values in the gene region of interest.

### PAML analysis

Multiple alignments were created with ClustalX2.0 [84]. Maximum likelihood analysis was performed with codeml in the PAML 4.1 software package [31]. To detect selection, multiple alignments were fitted to the NSsites models M1a (neutral model, codon values of dN/dS are fit into two site classes, one with value between 0 and 1, and one fixed at dN/dS = 1), M2a (positive selection model, similar to M1a but with an extra class of dN/dS>1 allowed), M7 (neutral model, codon values of dN/dS fit to a beta distribution, dN/dS>1 disallowed), M8a (neutral model, similar to M7 except with a fixed codon class of at dN/dS = 1) and M8 (positive selection model, similar to M7 but with an extra class of dN/dS>1 allowed). Simulations were run with multiple seed values for dN/dS (ω) and assuming either the f61 or f3x4 model of codon frequencies. Likelihood ratio tests were performed to assess whether permitting codons to evolve under positive selection gives a significantly better fit to the data (model comparisons M1a vs. M2a, M7 vs. M8, M8a vs. M8). In situations where the null model could be rejected (p<0.05), posterior probabilities were assigned to individual codons belonging to the class of codons with dN/dS>1. Residues under positive selection were mapped onto existing crystal structures using MacPyMol (v.0.99; http://pymol.sourceforge.net/).

The branch-site test allows identification of positive selection that might be limited to a subset of codons along only a subset of the branches being analyzed [34]. To implement this test, multiple alignments were fitted to the branch-sites Model A (positive selection model, codon values of dN/dS along background branches are fit into two site classes, one (ω_0_) between 0 and 1 and one (ω_1_) equal to 1, on the foreground branches a third site class is allowed (ω_2_) with dN/dS>1), and Model A with fixed ω_2_ = 1 (null model, similar to Model A except the foreground ω_2_ value is fixed at 1). Hominoids were defined as the “foreground” clade, with all other branches in the tree being defined as background branches. The likelihood of Model A is compared to the likelihood of the null model with a likelihood ratio test. Simulations were run with multiple seed values for dN/dS and assuming either the f61 or f3x4 models of codon frequencies. The “Fequal” codon model was also tested in the branch-site analysis of *NBS1*.

### Clustering analysis

To test the significance of clustering of the codons under positive selection in *XRCC4*, the statistical program R was utilized to perform a permutation test. The observed span of the positively selected codons on the primary sequence was compared with a null distribution created by calculating the span resulting from randomly generated sets of equivalent numbers of codons. We generated 100,000 random distances.

### Population genetic tests

To examine evidence for recent positive selection in humans, we implemented a previously published method that combines multiple tests for selection, the Composite of Multiple Signals (CMS) [45]. We have adapted the method to detect genomic regions under selection by examining the fraction of high scores in 100kb sliding windows. To determine the significance threshold, we used the *cosi* coalescent simulator to simulate 1,000 1MB autosomal regions, evolving neutrally under a previously validated demographic model [86]. We set thresholds that yielded a 0.1% false positive rate in simulations. Two long-haplotype tests, XP-EHH and iHS, were used to examine evidence for selection in or around the genes of interest. iHS was calculated as described in [10] for all SNPs with a minor allele frequency greater than 5%. iHS was analyzed independently in the European (CEU), East Asian (JPT and CHB), and West African (Yoruban; YRI) populations. XP-EHH was calculated as in [9] for the each of the three populations. For each SNP, we found the maximum score of the comparisons with the two other populations. In each 100kb window along the gene regions, the fraction of SNPs with |iHS|>2 or the maximum XP-EHH score was used as the test statistic. To calculate empirical P-values for each window *w*, we calculated the test statistics for each 100kb window across the genome and found the fraction of genomic windows with values of the test statistic greater than that found for window w. The ancestral state for each SNP was determined by comparison to the chimpanzee genome. We calculated Fst for each SNP in the regions using the Weir-Cockerham estimator [87]. Three pairwise comparisons were made between the African (Yoruban), European, and East Asian populations. For each population, we compared the allele frequency in that population to the average frequency in the other two populations. For each 100kb window across the region, the maximum Fst was used as the test statistic. To generate the null distribution, we performed the same procedure on each 100kb window in the genome and derived an empirical p-value based on this distribution.

### Plasmid constructs and expression

A biotinylated human MRN (E185) complex was expressed in a baculovirus system from the transfer vectors pTP11 (Rad50), pTP814 (Mre11), pTP1014 (Nbs1), and pTP1016 (BirA) as described earlier [88]. To make biotinylated human MRN (Q185) complex, the E to Q point mutation at Nbs1 position 185 was introduced into pTP994, whose bacmid form is pTP1014, by primer-based mutagenesis (QuikChange Kit, Invitrogen). Flag-tagged Mdc1 (amino acids 1–740) was expressed using bacmid construct pTP1188, which was made from the corresponding transfer vector pTP1187. Expression constructs for Flag-tagged and HA-tagged ATM were gifts from M. Kastan and R. Abraham. The E. coli expression construct for GST-p53 was described earlier [89].

### Protein purification

Purification procedures for the biotinylated MRN complex were the same as for the non-biotinylated MRN complex as described earlier [90]. Dimeric ATM was made by transient transfection of expression constructs into 293T cells using calcium phosphate and purified as described earlier [91]. Mdc1 (aa 1–740) was expressed in Sf21 insect cells using the Bac-to-Bac system (Invitrogen) and was purified identically to 53BP1 as described earlier [88]. The GST-p53 was purified identically to the GST–Brca1 fragments as described earlier [92] and was further purified by separation on a Superdex 200 gel filtration column (GE) in buffer A (100 mM NaCl, 25 mM Tris pH8, 10% glycerol, and 1 mM DTT). Protein concentrations were determined by quantification of protein preparations with standards on colloidal Coomassie-stained SDS–PAGE gels using the Odyssey system (LiCor).

### In vitro binding assay

20 nM biotinylated MRN complex was incubated with 45 nM Mdc1 (aa 1–740) in buffer A for 1 hour at 30°C in a final volume of 100 µl, then incubated with streptavidin-coated magnetic beads (Dynal) and 0.2% CHAPS (Sigma) while rotating at 4°C for 15 min. Beads with associated proteins were washed three times with buffer A containing 0.2% CHAPS, and bound proteins were eluted by boiling the beads in SDS loading buffer. Proteins were analyzed by SDS–PAGE and western blotting using antibodies directed against the Flag epitope (Sigma, F3165) and Nbs1 (Genetex, MSNBS10PX1).

### Kinase assay

ATM kinase assays were performed with 0.2 nM dimeric ATM, 50 nM GST–p53 substrate, and varying amounts of MRN complex (concentrations of MRN = 1.25, 2.5, 5, and 10 nM). Kinase assays were performed in kinase buffer (50 mM HEPES, pH 7.5, 50 mM potassium chloride, 5 mM magnesium chloride, 10% glycerol, 1 mM ATP, 1 mM DTT, and 10 ng DNA) for 90 min at 30°C in a volume of 40 microliters as described earlier [91]. Phosphorylated p53 (ser15) was detected as described earlier [91] using phospho-specific antibody from Calbiochem (PC461).

## Supporting Information

Figure S1 Sliding window analyses of all genes in the NHEJ pathway. The sliding window analysis of dN/dS along the length of each NHEJ gene is shown. In each case, three pairwise alignments were analyzed (human and orangutan comparison in pink, human and rhesus comparison in green, rhesus and marmoset comparison in orange). The maximum dN/dS value in each comparison was analyzed for statistical significance (dN/dS>1); an asterisk indicates statistically significant peaks (p<0.05).
(0.08 MB PDF)

Table S1 Primate samples used in study.
(0.03 MB PDF)

Table S2 Details of PCR and sequencing strategies.
(0.06 MB PDF)

Table S3 Primers used for amplification and sequencing of NHEJ genes.
(0.03 MB PDF)

Table S4 PAML analysis of primate NBS1 sequences.
(0.03 MB PDF)

Table S5 PAML analysis of primate CtIP sequences.
(0.03 MB PDF)

Table S6 PAML analysis of primate Artemis sequences.
(0.03 MB PDF)

Table S7 PAML analysis of primate XRCC4 sequences.
(0.03 MB PDF)

Table S8 PAML analysis of primate Polλ sequences.
(0.03 MB PDF)

Table S9 PAML analysis of primate XLF sequences.
(0.03 MB PDF)

Table S10 Branch-site test for positive selection in the hominoid clade for primate NHEJ genes.
(0.60 MB EPS)

Table S11 Variable codon models in NBS1 branch-site test for positive selection.
(0.50 MB EPS)

Table S12 Summary of human population genetic tests performed on HapMap data.
(0.04 MB PDF)
