# Supplementary figures and images for: Ancient and Recent Adaptive Evolution of Primate Non-Homologous End Joining Genes
Source: PLoS Genet. 2010 Oct 21;6(10):e1001169. doi: 10.1371/journal.pgen.1001169 (PMC2958818; doi:10.1371/journal.pgen.1001169)

Figure S1 - Sliding window analysis of human NHEJ genes

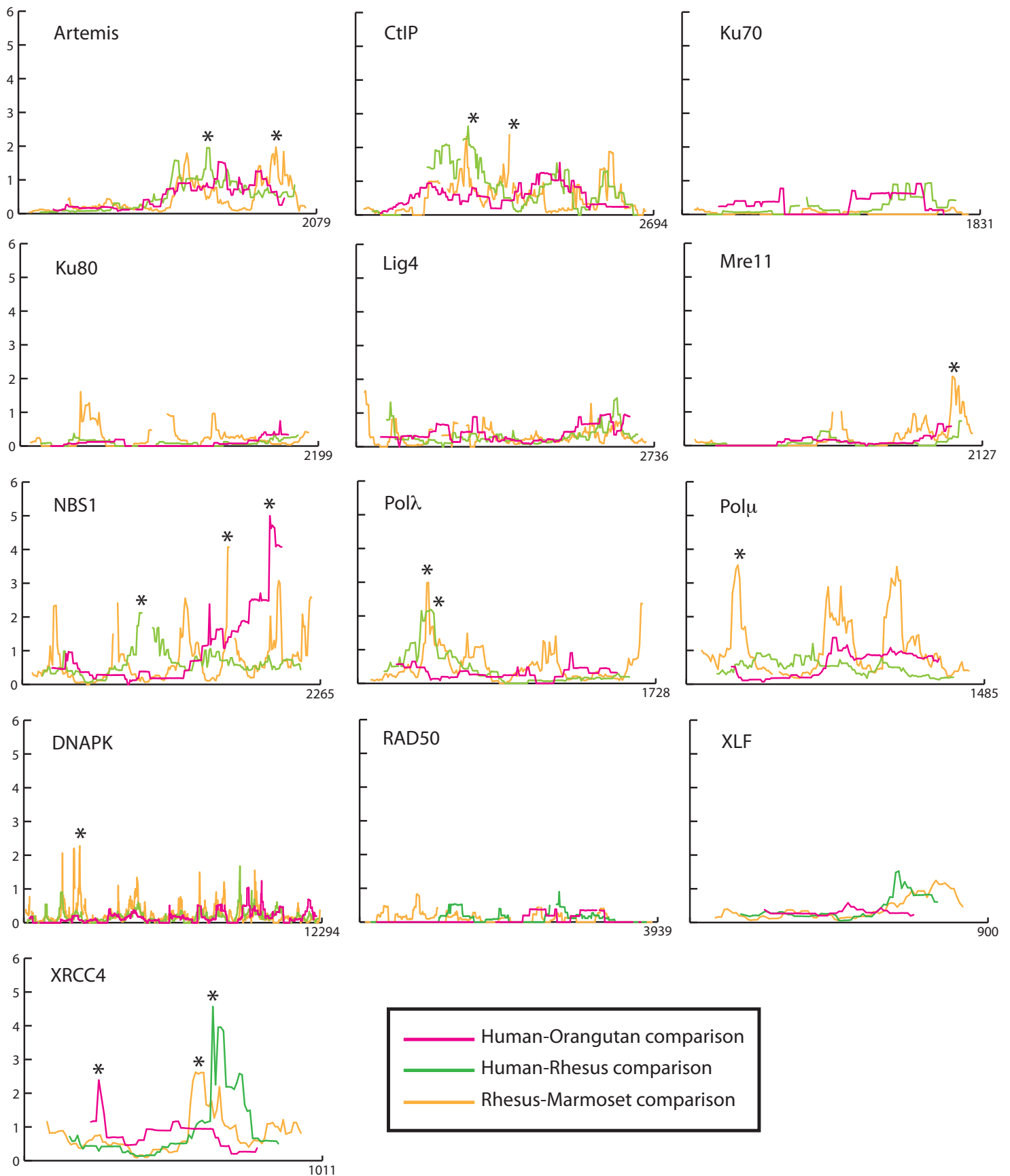

Supplement: Figure S1 — Sliding window analyses of all genes in the NHEJ pathway. The sliding window analysis of dN/dS along the length of each NHEJ gene is shown. In each case, three pairwise alignments were analyzed (human and orangutan comparison in pink, human and rhesus comparison in green, rhesus and marmoset comparison in orange). The maximum dN/dS value in each comparison was analyzed for statistical significance (dN/dS>1); an asterisk indicates statistically significant peaks (p<0.05). (0.08 MB PDF) [file pgen.1001169.s001.pdf]
