# Supplementary material for: Ancient and Recent Adaptive Evolution of Primate Non-Homologous End Joining Genes
Source: PLoS Genet. 2010 Oct 21;6(10):e1001169. doi: 10.1371/journal.pgen.1001169 (PMC2958818; doi:10.1371/journal.pgen.1001169)
Supplement: Table S1 — Primate samples used in study. (0.03 MB PDF) [file pgen.1001169.s002.pdf]

Table S1. Primate samples

Common Name		Source		Cell type
Gorilla	*Gorilla gorilla*	Coriell	PR00280	Fibroblasts
Sumatran Orangutan	*Pongo pygmaeus*	Coriell	PR01052	B-Lymphocyte
Borneo Orangutan	*Pongo pygmaeus*	Coriell	PR00650	B-Lymphocyte
Siamang	*Hylobates syndactylus*	Coriell	PR00722	Fibroblasts
White-Cheeked Gibbon	*Hylobates leucogeny*	Coriell	PR01037	Fibroblasts
Agile Gibbon	*Hylobates agilis*	Coriell	PR00773	Fibroblasts
Rhesus	*Macaca mulatta*	W. Johnson	Mm265-95	B-Lymphocyte
Talapoin	*Miopithecus talapoin*	Coriell	PR00716	Fibroblasts
Colobus	*Colobus guereza*	Coriell	PR00980	Fibroblasts
Leaf Monkey	*Trachypithecus francoisi*	Coriell	PR01099	Fibroblasts
Crab-eating Macaque	*Macaca fascicularis*	Coriell	103-06	B-Lymphocyte
Olive Baboon	*Papio anubis*	Coriell	PR00978	Fibroblasts
Black Mangabey	*Lophocebus albigena*	Coriell	PR01215	Fibroblasts
Wolf's Guenon	*Cercopithecus wolfi*	Coriell	PR01241	Fibroblasts
Mamoset	*Callithrix jacchus*	Coriell	GM07404	B-Lymphocyte
Squirrel Monkey	*Saimiri sciureus*	Coriell	PR00603	Fibroblasts
Howler Monkey	*Alouatta sara*	Coriell	PR00708	Fibroblasts
Titi Monkey	*Callicebus cupreus*	Coriell	PR00793	Fibroblasts
