# Supplementary material for: Ancient and Recent Adaptive Evolution of Primate Non-Homologous End Joining Genes
Source: PLoS Genet. 2010 Oct 21;6(10):e1001169. doi: 10.1371/journal.pgen.1001169 (PMC2958818; doi:10.1371/journal.pgen.1001169)
Supplement: Table S2 — Details of PCR and sequencing strategies. (0.06 MB PDF) [file pgen.1001169.s003.pdf]

Table S2. NHEJ sequencing
This table lists PCR and sequencing primers used to amplify and sequence primate NHEJ genes from cDNA or mRNA
(PCR primers), *sequencing primer
Bold=fragment cloned into TA vector before sequencing, >3 clones sequenced
All others sequenced directly from PCR product pool

Artemis

Gorilla	(AD45/AD241) AD45*, AD93*<br>(AD238/AD241) AD243*, AD238*, AD241*<br>(AD52/AD55) AD240*, AD52*, AD94*, AD251*
Borneo Orangutan	**(AD238/AD55)** M13F*, AD93*, AD243*, AD241*, M13R*
Siamang	(AD45/AD241) AD93*, AD45*<br>(AD238/AD24) AD243*, AD238*, AD95*, AD52*<br>(AD52/AD55) AD51*, AD94*<br>(AD48/AD55) AD55*
White-Cheeked Gibbon	(AD238/AD241) AD93*, AD243*, AD238*, AD241*<br>(AD45/AD241) AD45*<br>(AD51/AD55) AD51*, AD55*, AD251*
Agile Gibbon	(AD45/AD241) AD45*<br>(AD238/AD241) AD238*, AD243*, AD241*, AD52*<br>(AD51/AD53) AD94*, AD251*
Talapoin	(AD46/AD242) AD46*<br>(AD239/AD240) AD239*, AD93*, AD95*, AD52*, AD94*<br>(AD46/AD49) AD50*<br>(AD48/AD55) AD55*
Colobus	(AD46/AD242) AD46*<br>(AD239/AD242) AD239*, AD242*<br>(AD239/AD240) AD52*, AD94*<br>(AD52/AD240) AD52*
Leaf Monkey	(AD52/AD55) AD52*<br>(AD46/AD49) AD50*<br>(AD239/AD242) AD242*<br>**(AD239/AD55)** M13F*, AD243*, AD241*, M13R*
Crab-eating Macaque	(AD46/AD242) AD46*<br>(AD239/AD240) AD93*, AD239*, AD243*, AD95*, AD52*, AD94*<br>(AD46/AD49) AD50*<br>(AD48/AD55) AD49*, AD52*
Olive Baboon	(AD46/AD242) AD46*<br>(AD239/AD242) AD93*, AD239*, AD243*, AD95*, AD52*<br>(AD52/AD55) AD51*, AD94*
Black Mangabey	(AD46/AD50) AD46*, AD243*<br>(AD239/AD242) AD239*, AD93*, AD95*, AD52*<br>(AD52/AD55) AD51*, AD94*
Wolf's Guenon	(AD45/AD242) AD252*<br>(AD239/AD242) AD239*, AD95*, AD242*<br>(AD239/AD240) AD93*, AD243*, AD52*, AD94*
Squirrel Monkey	(AD46/AD50) AD93*, AD46*, AD95*<br>(AD52/AD55) AD49*, AD52*, AD51*, AD55*
Howler Monkey	(AD49/AD137) AD93*, AD47*, AD95*, AD241*, AD94*<br>(AD52/AD55) AD52*, AD51*, AD55*
Titi Monkey	**(AD238/AD55)** M13F*, M13R*, AD241*<br>(AD95/AD137) AD95*, AD51*, AD55*, AD94*

CtIP

Gorilla	(AD57/AD59) AD57*, AD96*, AD59*<br>(AD62/AD66) AD62*, AD97*, AD66*, AD68*
Sumatran Orangutan	(AD57/AD59) AD56*<br>(AD62/AD65) AD66*
Borneo Orangutan	(AD57/AD59) AD193*, AD56*, AD59*<br>(AD62/AD65) AD62*, AD97*, AD65*
Siamang	(AD57/AD59) AD56*, AD57*, AD59*<br>(AD62/AD66) AD62*, AD97*, AD66*, AD68*
White-Cheeked Gibbon	(AD57/AD59) AD57*, AD56*, AD96*, AD59*, AD62*, AD68*, AD65*<br>(AD62/AD67) AD61*, AD64*
Agile Gibbon	(AD57/AD59) AD57*, AD59*<br>(AD62/AD67) AD62*, AD97*<br>(AD61/AD65) AD68*
Talapoin	(AD56/AD59) AD56*, AD59*<br>(AD62/AD65) AD61*, AD97*, AD65*
Colobus	(AD56/AD59) AD56*, AD59*<br>(AD62/AD65) AD62*, AD97*, AD68*
Leaf Monkey	(AD56/AD59) AD56*, AD96*, AD59*<br>(AD62/AD65) AD61*, AD97*, AD68*, AD65*
Rhesus	(AD62/AD64) AD62*, AD64*
Crab-eating Macaque	(AD56/AD67) AD56*, AD61*, AD68*<br>(AD56/AD59) AD56*, AD59*<br>(AD62/AD65) AD62*, AD64*, AD65*, AD68*
Olive Baboon	(AD57/AD59) AD57*, AD59*, AD96*<br>(AD62/AD65) AD62*, AD97*, AD64*, AD68*, AD65*
Black Mangabey	(AD56/AD59) AD56*, AD59*, AD96*<br>(AD62/AD67) AD62*, AD97*<br>(AD61/AD67) AD61*, AD67*, AD68*
Wolf's Guenon	(AD56/AD59) AD56*, AD59*, AD96*<br>(AD62/AD65) AD97*, AD61*, AD64*, AD66*, AD65*, AD68*
Marmoset	(AD58/AD60) AD56*<br>(AD56/AD65) AD64*
Squirrel Monkey	(AD56/AD65) AD56*, AD59*, AD61*, AD64*, AD65*, AD68*
Howler Monkey	(AD56/AD65) AD56*, AD60*, AD63*, AD97*, AD61*, AD68*, AD65*
Titi Monkey	(AD58/AD60) AD193*, AD60*, AD96*<br>(AD61/AD65) AD195*, AD97*, AD64*, AD68*

XLF

Gorilla	(AE85/AE87) AE85*, AE87*<br>(AE86/AE89) AE86*, AE89*
Borneo Orangutan	(AE86/AE89) AE87*, AE83*, AE89*, AD35*
Siamang	(AE85/AE87) AE85*, AE87*, AE180*<br>(AE86/AE89) AE88*, AE181*
White-Cheeked Gibbon	(AE85/AE87) AE85*, AE87*<br>(AE86/AE89) AE86*, AD35*, AD36*, AE89*
Agile Gibbon	(AE85/AE87) AE85*, AE87*<br>(AE86/AE89) AD36*, AE86*, AE89*
Talapoin	(AE85/AE87) AE87*, AE180*, AE187*<br>(AE86/AE89) AE85*, AE88*, AE89*, AE181*
Colobus	(AE85/AE87) AE87*, AE180*<br>(AE86/AE89) AE85*, AE88*, AE181*
Leaf Monkey	(AE85/AE87) AE85*, AE87*, AE186*<br>(AE86/AE89) AE88*, AE181*
Crab-eating Macaque	(AE85/AE87) AE85*, AE87*<br>(AE86/AE89) AE86*, AE89*
Olive Baboon	(AE85/AE87) AE85*, AE87*<br>(AE86/AE89) AE86*, AE89*
Black Mangabey	(AE85/AE87) AE85*, AE87*<br>(AE86/AE89) AE86*, AE89*
Wolf's Guenon	(AE85/AE87) AE85*, AE87*<br>(AE86/AE89) AE86*, AE89*
Squirrel Monkey	(AE85/AE87) AE85*, AE87*<br>(AE86/AE89) AE86*, AE89*
Howler Monkey	(AE85/AE87) AE85*, AE87*<br>(AE86/AE89) AE86*, AE89*
Titi Monkey	(AE85/AE87) AE85*, AE87*<br>(AE86/AE89) AE86*, AE89*

XRCC4

Gorilla	(AE129/AE131) AE129*, AE131*<br>(AE130/AE24) AE130*, AE24*
Sumatran Orangutan	(AE129/AE131) AE131*<br>(AE130/AE24) AE130*
Borneo Orangutan	(AE129/AE131) AE129*, AE131*<br>(AE130/AE24) AE130*, AE24*
Siamang	(AE129/AE131) AE129*, AE131*<br>(AE130/AE24) AE132*, AE173*
White-Cheeked Gibbon	(AE129/AE131) AE129*, AE131*<br>(AE130/AE24) AE130*, AE24*
Agile Gibbon	(AE129/AE131) AE129*, AE131*<br>(AE130/AE24) AE130*, AE132*
Talapoin	(AE129/AE131) AE129*, AE131*, AE179*<br>(AE130/AE24) AE132*, AE173*
Colobus	(AE129/AE131) AE131*, AE172*, AE178*<br>(AE130/AE24) AE132*, AE130*
Leaf Monkey	(AE66/AE24) AE131*, AE172*, AE178*, AE179*<br>(AE129/AE131) AE131*, AE172*, AE178*<br>(AE178/AE172) AE172*, AE178*<br>(AE130/AE24) AE130*, AE132*, AE173*
Crab-eating Macaque	(AE129/AE131) AE129*, AE131*<br>(AE130/AE24) AE130*, AE132*
Olive Baboon	(AE129/AE131) AE129*, AE131*<br>(AE130/AE24) AE130*, AE24*
Black Mangabey	(AE129/AE131) AE129*, AE131*<br>(AE130/AE24) AE130*, AE24*
Wolf's Guenon	(AE129/AE131) AE129*, AE131*<br>(AE130/AE24) AE130*, AE24*
Marmoset	(AE130/AE24) AE130*
Squirrel Monkey	(AE66/AE132) AE66*, AE131*, AE130*, AE132*
Howler Monkey	(AE129/AE131) AE129*, AE131*<br>(AE66/AE132) AE131*, AE66*, AE130*
Titi Monkey	(AE66/AE24) AE131*, AE66*, AE130*, AE132*<br>(AE130/AE24) AE130*, AE24*

NBS1

Gorilla	(AE133/AE136) AD1*, AE133*, AE136*, AD2*<br>(AE182/AE138) AD3*, AE138*, AE182*<br>(AE137/AE169) AE137*, AE169*
Sumatran Orangutan	(AD2/AE138) AD2*<br>(AE137/AE169) AE137*
Borneo Orangutan	(AE133/AE136) AD1*, AE136*, AE133*, AD2*<br>(AE182/AE138) AE182*, AE138*<br>(AE137/AE169) AE137*, AD4*, AE169*
Siamang	(AE133/AE136) AE133*, AE134*, AE136*, AE183*<br>(AE182/AE171) AE182*, AE171*, AE137*, AE138*<br>(AE135/AE138) AE135*<br>(AE135/AE136) AE135*<br>(AE182/AE138) AE182*<br>(AE137/AE185) AE184*, AE185*<br>(AE137/AE169) AE169*, AE185*
White-Cheeked Gibbon	(AE133/AE136) AD1*, AE133*, AE136*, AD2*<br>(AE182/AE138) AD3*, AE182*, AE138*<br>(AE135/AE136) AE135*, AE169*<br>(AE184/AE139) AE185*, AE184*, AE169*, AE139*<br>(AD4/AE169) AE185*, AE169*, AD4*
Agile Gibbon	(AE133/AE136) AD1*, AE133*, AE136*, AD2*<br>(AE182/AE138) AD3*, AE138*, AE182*<br>(AE137/AE185) AE137*, AE185*<br>(AE137/AE169) AE137*, AE169*, AD4*
Talapoin	(AE133/AE136) AE133*, AE136*<br>(AE182/AE138) AE138*, AE182*<br>(AE184/AE185) AE184*, AE185*<br>(AE182/AE169) AE137*, AE169*, AE182*, AE185*<br>(AE137/AE169) AE137*, AE169*, AE184*
Colobus	(AE133/AE136) AE133*, AE183*<br>(AE135/AE138) AE135*, AE137*, AE182*<br>(AE134/AE139) AE136*<br>(AE137/AE169) AE137*, AE169*
Leaf Monkey	(AE133/AE136) AE134*, AE136*, AE183*<br>(AE135/AE138) AE135*, AE138*<br>(AE134/AE139) AE135*, AE137*, AE138*, AE184*, AE185*
Crab-eating Macaque	(AE133/AE136) AD1*, AE133*, AE136*, AD2*<br>(AE182/AE169) AD3*<br>(AE182/AE138) AE182*, AE138*<br>(AE137/AE169) AE137*, AE169*, AD4*
Olive Baboon	(AE133/AE136) AD1*, AE133*, AE136*<br>(AD2/AE169) AD3*, AE182*, AE185*, AE169*, AD4*
Black Mangabey	(AE133/AE136) AD1*, AE136*, AE133*<br>(AE182/AE169) AD3*, AE182*, AE169*
Wolf's Guenon	(AE133/AE136) AD1*, AE133*, AE136*<br>(AE182/AE169) AD3*, AE182*, AE169*, AD4*
Marmoset	(AD2/AE170) AE138*, AE185*
Squirrel Monkey	(AE133/AE136) AE133*, AE136*, AD1*, AD2*<br>(AE135/AE138) AE135*, AE138*, AD3*<br>(AE137/AE170) AE137*, AE170*, AD4*
Howler Monkey	(AE133/AE136) AE133*, AE136*, AD1*, AD2*<br>(AE135/AE138) AE135*, AE138*, AD3*<br>(AE137/AE171) AE140*, AD4*, AE184*, AE185*
Titi Monkey	(AE133/AE136) AD1*, AE133*, AE136*<br>(AD2/AE138) AD2*, AE138*<br>(AE137/AE170) AE137*, AE170*

PolL

Gorilla	(**SS73/AD187**) AD189*, AD260*, AD188*<br>(SS73/AD189) AD189*<br>(SS73/AD187) AD189*, SS73*, AD187*<br>(AD188/SS75) SS84*, AD187*, AD188*, SS81*, AD190*<br>(SS72/SS74) SS74*
Sumatran Orangutan	(**SS73/AD187**) AD189*, AD260*, AD188*<br>(AD188/SS75) SS84*, SS81*<br>(SS72/SS74) SS74*
Borneo Orangutan	(**SS73/SS75**) AD189*, AD260*, AD187*, AD188*
Siamang	(**SS73/AD187**) AD189*, SS80*, AD260*, AD188*<br>(**AD188/SS75**) SS84*, SS81*<br>(AD188/AD256) SS84*, SS81*
White-Cheeked Gibbon	(**SS73/AD187**) AD189*, AD260*, AD188*<br>(**AD188/SS75**) AD187*, SS78*<br>(AD188/SS75) SS84*, SS81*, SS78*<br>(SS72/SS74) SS74*
Agile Gibbon	(**SS73/AD187**) AD189*, AD260*, AD188*<br>(**AD188/SS75**) SS78*, AD187*<br>(AD188/SS75) SS78*, SS81*, SS84*<br>(SS72/SS74) SS74*
Talapoin	(**SS73/AD187**) AD189*, AD260*, AD188*<br>(AD188/SS75) SS81*, SS84*
Colobus	(**SS73/AD187**) AD189*, AD260*, AD188*<br>(SS72/SS74) SS74*<br>(AD188/SS75) SS84*, SS78*, SS81*
Leaf Monkey	(**SS72/SS74**) AD189*, AD187*, AD188*, SS78*<br>(**SS73/AD187**) AD189*, AD260*<br>(AD188/SS75) SS78*, SS81*, SS84*<br>(SS72/SS74) AD187*
Crab-eating Macaque	(**SS73/AD187**) AD189*, AD260*, AD188*<br>(SS73/AD187) AD189*<br>(SS73/AD189) AD189*<br>(SS72/SS74) AD187*<br>(AD188/SS75) SS84*, AD187*, AD188*, SS78*, SS81*, AD190*
Olive Baboon	(**SS73/AD187**) AD189*, AD260*, AD188*<br>(SS72/SS74) AD187*, AD188*<br>(AD188/SS75) SS81*, SS84*, SS78*
Black Mangabey	(**SS73/AD187**) AD189*, AD260*, AD188*<br>(AD188/SS75) SS84*, SS78*, SS81*<br>(SS72/SS74) SS74*
Wolf's Guenon	(**SS73/AD187**) AD189*, AD260*, AD188*<br>(AD188/SS75) SS81*, SS84*
Squirrel Monkey	(**SS73/AD187**) AD189*, AD260*, SS80*, AD188*, SS78*<br>(AD188/SS75) SS84*, SS81*, SS78*
Howler Monkey	(**SS73/AD187**) AD189*, AD260*, AD188*<br>(AD188/SS75) SS81*, SS84*
Titi Monkey	(**SS73/AD187**) AD189*, AD260*, AD188*<br>(SS72/SS74) AD187*, AD188*<br>(AD188/SS75) SS84*, SS78*, SS81*
