# Supplementary material for: Ancient and Recent Adaptive Evolution of Primate Non-Homologous End Joining Genes
Source: PLoS Genet. 2010 Oct 21;6(10):e1001169. doi: 10.1371/journal.pgen.1001169 (PMC2958818; doi:10.1371/journal.pgen.1001169)
Supplement: Table S3 — Primers used for amplification and sequencing of NHEJ genes. (0.03 MB PDF) [file pgen.1001169.s004.pdf]

Table S3. Primers used for amplification and sequencing of NHEJ genes

Primer Name	Sequence
AD001	GCAAACACTCCAAAGGTAATACTATCCC
AD002	CACCTTGTCATGGTATCAGTTAAAGTTACC
AD003	GGGTGTAGCAGGTTGTGTTTGTTC
AD004	TTGGAACAGTTATTCAAGGACACAAAACC
AD035	CCTGGTCTCCCAACATTTGATTCG
AD036	CGAATCAAATGTTGGGAGACCAGG
AD045	TCAGGGCTGGCCTTGGCTTC
AD046	TCTGGGCTGGCCTGGGTTTC
AD047	CGTTGGTATCGGTGGCACTATG
AD048	TTGGGATCGGCGGCGATATG
AD049	GTACATCCCCATCAGCCTTTTGC
AD050	TGTGCCTTAAAGGTATTGGCAGAGG
AD051	TATAAACCACTGGGAAAACTGAAGAGAGC
AD052	CAAATGTAATTGTGAGGACTGGAGAGAG
AD055	TGCAGGTTTTTTAGTGGTTGCTCTAGG
AD056	ACCTGTCCAAAGACGACTTGATACC
AD057	CTTCCCTTCGGGGGCTTTCG
AD058	TCGGGGGCTTCCGCAAATCC
AD059	GCAGCTTACTTCATGTTCACTTTCTTCC
AD060	GGCAGTTTACTTCAAGTTCACTTTCTTCC
AD061	AAACTAGATCAAAATCTGAAGATAGTGCCC
AD062	TTCAAAGACTCCTCCTCAAGAAGAATTACC
AD063	TTCAAAGACTCCTCCTCAAGAAGAATTGCC
AD064	GGTGTCTTGAGCAGGAAGCC
AD065	TTAAAGGTGCAAAAGCAAAATATCACAAAC
AD066	CTTCATCCTTCTGTTTCTGTTTCAACGTCTA
AD067	CTCCATCCTTCTGTTTCTTTTTCAACATCTA
AD068	AGTATAGATCCGGGAGCAGACC
AD093	CTTAAACACTCCTCCCGACTTGG
AD094	CAGTGACTCTGATGGAGAATC
AD095	CCAAGTCGGGAGGAGTGTTTAAG
AD096	GAGGAAGACGTTATTCCAGATTCACC
AD097	TTTTCTCCCTTCTGCTCTTGC
AD137	CCTCTGTCTTCTGGGCTCAAGTG
AD187	ACTGTCTGCTCAATCTCTGTAGCC
AD188	ATTGGGAAGCGGATGGCTGAG
AD189	CTAACCTGGGTTTCTTCCCCATC
AD190	TCATCACTCACCCAGATGGCC
AD193	GATTATGTTGTGGATGAGTTGAAGACTTGG
AD195	CAAAGCCTCATAAAGAGTCACTTGCC
AD238	TATGAGTTCTTTCGAGGGGCAGATG
AD239	TATGAGTTCCTTCGAGGGGCAGAT
AD240	TGGTTGCTCTAGGTTGAAACGCTTTGAAT
AD241	TCCCAGCCTTGACTTCCTTGTTC
AD242	CCAGCCTTGACTTCCGTGTTC
AD243	GCTCTCTTCAGTTTTCCCAGTGGTTTATA
AD251	GCTGACTACAGACCAACAATCAAAG
AD252	CTCCAACCTTCTTTTCAAGGTAGG
AD256	TCTCCTCAGCACCCCCAGC
AD260	CCAGCTCCCCAGATGTTGGA
AE024	GATGTTTTCTTTGTCCTGTTTTCAAAATCTTCC

AE066	GAAGTAGCTGATACTCTCATTGGTTGC
AE083	GCG TGC GGC TAA GAG AGT GG
AE085	GAT GGA GGA ACT GGA GCA AGG
AE086	GAG CCA AGG AGC TGA ACA AGC
AE087	CCT GGT AGT CTT GGA TCT CTA GG
AE088	GAA GCT GTT CTC CAA GTC CAT CC
AE089	CAG AAC CTC CAC CAA AAG AGA GG
AE129	GAT ACC GGA AGT AGA GTC ACG G
AE130	CAT TAG AGA ACT TAT TTG TTA TTG CTT GGA CAC C
AE131	ATC ATA GAC TGG ATC TCG GTC AGC
AE132	GTG TGA ATG AAT ATG ATA AAT GTG TCC TTT GC
AE133	CGT ATC CGC GCT AGT CTA CC
AE134	GTT ACG CGG TTG CAC GTC G
AE135	GCA GTT GTC TTT GGA GGT GGG
AE136	GTC TTT AAT CCT GTA CTG GGA TGG C
AE137	GAA CAA ACA CAA CCT GCT ACA CCC
AE138	CTG GTT TTG TGT CCT TGA ATA ACT GTT CC
AE139	GCT AGG ACA ATG GTG GAA GGG
AE140	GCT AGG ATA GTG GTG GAA GGG
AE169	ACC TTG TTG GCC TGA AGT AGA TGC
AE170	GGT AGC CTG AAG TAG ATG CCT GC
AE171	GCA CCA CTG AAG CCA TTT TGT TTG G
AE172	GCT TGA TTT TCT GCG ATG GTG TCC
AE173	GGA GCC ATT TTA GGT TCT GTC CC
AE178	CTT GGG AGA AAA CAC TGG AAT CTG G
AE179	CCT GCT CCT GAC ACC AAT GC
AE180	CCA CCT GTT CAT GCC ACA CC
AE181	CAG CCA GAA CAA CTG GTC TCC
AE182	CGG AAA CGT GTG TTG TTG ATA CAG G
AE183	CGA CTT CAA AGT TCG GGA AGA GC
AE184	GAG CAG CAT CTA TCT CAG AAT GAG C
AE185	CCT CCA TTT CCT GCC TTA GCC
AE186	CTT GTT CAG CTC CTT GGC TCG
AE187	ATG GTC ATT CAG CGT GGA CTG G
ss072	GGATGCAGCCGTACTTCAATGG
ss073	GATCTAGCCGTACTTCAATGGATCCC
ss074	TCACCAGTCCCGCTCAGCAGG
ss075	CAGCCAGCTGAGGCTGGAGGG
ss078	TCCAACATCTGGGGAGCTGG
ss080	GATCCCAGGGGTATCTTGAAGG
ss081	AGTCCAGAAAGCAGCCCAGG
ss084	CACCAAGTCATCTGTGAGGAACC
