# Supplementary material for: Ancient and Recent Adaptive Evolution of Primate Non-Homologous End Joining Genes
Source: PLoS Genet. 2010 Oct 21;6(10):e1001169. doi: 10.1371/journal.pgen.1001169 (PMC2958818; doi:10.1371/journal.pgen.1001169)
Supplement: Table S5 — PAML analysis of primate CtIP sequences. (0.03 MB PDF) [file pgen.1001169.s006.pdf]

Table S5. PAML analysis of primate CtIP sequences.

CtIP dataset[a]	ω_0[b]	codon freq.[c]	*M1a-M2a*		*M7-M8*		*M8a-M8*		tree length[e]	dN/dS (%)[f]	AA Positions of dN/dS>1[g] * p>0.95 ** p>0.99	
			$2\Delta\ell$[d]	p-value	$2\Delta\ell$[d]	p-value	$2\Delta\ell$[d]	p-value			NEB	BEB
19 primate dataset	0.4	f61	8.9	p<0.02	10.0	p<0.007	8.4	p<0.004	0.52	2.11 (14.8%)	155, 187*, 235, 333*, 336, 355**, 365*, 368, 399, 416*, 420*, 425**, 481, 486*, 515*, 541**, 544, 554, 574*, 605*, 619, 724, 730	333, 355*, 365, 416, 425*, 486, 541*
	0.4	f3x4	4.9	p=0.087	5.4	p=0.066	4.9	p<0.028	0.50	2.37 (7.0%)	355*, 541	355*, 425, 541*
	1.6	f61	8.9	p<0.02	10.9	p<0.005	8.4	p<0.004	0.52	2.11 (14.8%)	155, 187*, 235, 333*, 336, 355**, 365*, 368, 399, 416*, 420*, 425**, 481, 486*, 515*, 541**, 544, 554, 574*, 605*, 619, 724, 730	333, 355*, 365, 416, 425*, 486, 541*
	1.6	f3x4	4.9	p=0.087	5.4	p=0.066	4.9	p<0.028	0.50	2.37 (7.0%)	355*, 541	355*, 425, 541*

a Dataset consisted of the aligned primate sequences *Homo sapiens, Pan troglodytes, Gorilla gorilla, Pongo pygmaeus* (Sumatran Orangutan), *Pongo pygmaeus* (Borneo Orangutan), *Hylobates syndactylus, Hylobates leucogenys, Hylobates agilis, Macaca mulatta, Macaca fascicularis, Lophocebus albigena, Papio anubis, Miopithecus talapoin, Cercopithecus wolfi, Colobus guereza, Trachypithecus francoisi, Saimiri sciureus, Callithrix jacchus,* and *Alouatta sara*.

b Initial seed value for ω (dN/dS) used in the maximum likelihood simulation

c Model of codon frequency

d Twice the difference in the natural logs of the likelihoods ($\Delta\ell$ x 2) of the two models being compared. This value is used in a likelihood ratio test along with the degrees of freedom. In all cases (M1a-M2a), (M7-M8), (M8a-M8), a model that allows positive selection is compared to a null model. The p-value indicates the confidence with which the null model can be rejected.

e The tree length is the number of substitutions per site along all branches in the phylogeny. It is calculated as the sum of the branch lengths, and is a representation of total diversity in the dataset

f dN/dS value of the class of codons evolving under positive selection in M8, and the percent of codons falling in that class.

g Amino acid positions identified in the class of codons evolving under positive selection in M8 with a posterior probability >0.90. Coordinates correspond to the human protein.
