# Supplementary material for: Ancient and Recent Adaptive Evolution of Primate Non-Homologous End Joining Genes
Source: PLoS Genet. 2010 Oct 21;6(10):e1001169. doi: 10.1371/journal.pgen.1001169 (PMC2958818; doi:10.1371/journal.pgen.1001169)
Supplement: Table S6 — PAML analysis of primate Artemis sequences. (0.03 MB PDF) [file pgen.1001169.s007.pdf]

Table S6. PAML analysis of primate Artemis sequences.

Artemis dataset[a]	ω_0[b]	codon freq.[c]	*M1a-M2a* 2Δℓ[d]	p-value	*M7-M8* 2Δℓ[d]	p-value	*M8a-M8* 2Δℓ[d]	p-value	tree length[e]	dN/dS (%)[f]	AA Positions of dN/dS>1[g] * p>0.95 ** p>0.99 NEB	BEB
All 20 primates	0.4	f61	3.4	p=0.181	5.1	p=0.077	3.4	p=0.064	0.59	1.84 (11.4%)		
	0.4	f3x4	2.7	p=0.254	5.0	p=0.082	2.7	p=0.098	0.59	1.77 (10.5%)		
	1.6	f61	3.4	p=0.181	5.1	p=0.077	3.4	p=0.064	0.59	1.84 (11.4%)		
	1.6	f3x4	2.7	p=0.254	5.0	p=0.082	2.7	p=0.098	0.59	1.77 (10.5%)		
18 primate subset	0.4	f61	5.4	p=0.066	7.2	p<0.03	5.5	p<0.02	0.59	2.06 (10.5%)	83*, 250**, 365*, 411*, 418**, 439*, 463*, 484*, 503*, 511, 576**, 610*, 626*	250, 418, 576
	0.4	f3x4	4.4	p=0.112	6.8	p<0.04	4.4	p<0.04	0.59	1.93 (10.2%)	83, 250**, 365, 411*, 418**, 439*, 463*, 484*, 503, 511, 576**, 610*, 626	
	1.6	f61	5.4	p=0.066	7.2	p<0.03	5.5	p<0.02	0.59	2.06 (10.5%)	83*, 250**, 365*, 411*, 418**, 439*, 463*, 484*, 503*, 511, 576**, 610*, 626*	250, 418, 576
	1.6	f3x4	4.4	p=0.112	6.8	p<0.04	4.4	p<0.04	0.59	1.93 (10.2%)	83, 250**, 365, 411*, 418**, 439*, 463*, 484*, 503, 511, 576**, 610*, 626	

[a] Dataset consisted of the aligned primate sequences *Homo sapiens, Pan troglodytes, Gorilla gorilla, Pongo pygmaeus* (Sumatran Orangutan), *Pongo pygmaeus* (Borneo Orangutan), *Hylobates syndactylus, Hylobates leucogenys, Hylobates agilis, Macaca mulatta, Macaca fascicularis, Lophocebus albigena, Papio anubis, Miopithecus talapoin, Cercopithecus wolfi, Colobus guereza, Trachypithecus francoisi, Saimiri sciureus, Callithrix jacchus, Callicebus cupreus,* and *Alouatta sara.* Eighteen primate subset consists of all primates listed above except *Lophocebus albigena* and *Papio anubis* due to a gap in both primates sequence.

[b] Initial seed value for ω (dN/dS) used in the maximum likelihood simulation

[c] Model of codon frequency

[d] Twice the difference in the natural logs of the likelihoods (Δℓ x 2) of the two models being compared. This value is used in a likelihood ratio test along with the degrees of freedom. In all cases (M1a-M2a), (M7-M8), (M8a-M8), a model that allows positive selection is compared to a null model. The p-value indicates the confidence with which the null model can be rejected.

[e] The tree length is the number of substitutions per site along all branches in the phylogeny. It is calculated as the sum of the branch lengths, and is a representation of total diversity in the dataset

[f] dN/dS value of the class of codons evolving under positive selection in M8, and the percent of codons falling in that class.

[g] Amino acid positions identified in the class of codons evolving under positive selection in M8 with a posterior probability >0.90. Coordinates correspond to the human protein.
