# Supplementary material for: Ancient and Recent Adaptive Evolution of Primate Non-Homologous End Joining Genes
Source: PLoS Genet. 2010 Oct 21;6(10):e1001169. doi: 10.1371/journal.pgen.1001169 (PMC2958818; doi:10.1371/journal.pgen.1001169)
Supplement: Table S7 — PAML analysis of primate XRCC4 sequences. (0.03 MB PDF) [file pgen.1001169.s008.pdf]

Table S7. PAML analysis of primate XRCC4 sequences.

XRCC4 [a] dataset	ω_0 [b]	codon freq. [c]	M1a-M2a 2Δℓ [d]	M1a-M2a p-value	M7-M8 2Δℓ [d]	M7-M8 p-value	M8a-M8 2Δℓ [d]	M8a-M8 p-value	tree length [e]	dN/dS (%) [f]	AA Positions of dN/dS>1 [g] * p>0.95 ** p>0.99 NEB	BEB
Full length protein	0.4	f61	8.7	p<0.014	8.7	p<0.013	8.5	p<0.004	0.62	15.36 (0.6%)	205 (0.79), 243**	205*, 211 (0.80), 216 (0.80), 218(0.85) , 243*, 292 (0.63)
	0.4	f3x4	9.8	p<0.008	10.0	p<0.007	10.0	p<0.002	0.62	16.7 (0.6%)	205 , 243**	205*, 211 (0.79), 216 (0.71), 218(0.76) , 243*, 292 (0.62)
	1.6	f61	8.7	p<0.014	8.7	p<0.013	8.5	p<0.004	0.62	15.4 (0.6%)	205 (0.79), 243**	205*, 211 (0.80), 216 (0.80), 218(0.85) , 243*, 292 (0.63)
	1.6	f3x4	9.8	p<0.008	10.0	p<0.007	10.0	p<0.002	0.62	16.7 (0.6%)	205 , 243**	205*, 211 (0.79), 216 (0.71), 218(0.76) , 243*, 292 (0.62)
C-terminal domain (aa204-336	0.4	f61	21.1	p<0.001	21.4	p<0.001	21.1	p<0.001	0.84	8.7 (8.1%)	205**, 211*, 216*, 218**, 243**, 292	205*, 211, 216, 218*, 243**, 292 (0.85)
	0.4	f3x4	13.1	p<0.002	13.4	p<0.002	13.1	p<0.001	0.76	6.1 (6.9%)	205**, 211*, 216, 218*, 243**, 292(0.85)	205*, 211 (0.80), 216 (0.68), 218(0.79), 243**, 292(0.67)
	1.6	f61	21.1	p<0.001	21.4	p<0.001	21.1	p<0.001	0.84	8.7 (8.1%)	205**, 211*, 216*, 218**, 243**, 292	205*, 211, 216, 218*, 243**, 292 (0.85)
	1.6	f3x4	13.1	p<0.002	13.4	p<0.002	13.1	p<0.001	0.76	6.1 (6.9%)	205**, 211*, 216, 218*, 243**, 292(0.85)	205*, 211 (0.80), 216 (0.68), 218(0.79), 243**, 292(0.67)

[a] Dataset consisted of the aligned primate sequences *Homo sapiens, Pan troglodytes, Gorilla gorilla, Pongo pygmaeus* (Sumatran Orangutan), *Pongo pygmaeus* (Borneo Orangutan), *Hylobates syndactylus, Hylobates leucogenys, Hylobates agilis, Macaca mulatta, Macaca fascicularis, Lophocebus albigena, Papio anubis, Miopithecus talapoin, Cercopithecus wolfi, Colobus guereza, Trachypithecus francoisi, Saimiri sciureus, Callithrix jacchus, Callicebus cupreus,* and *Alouatta sara.*

[b] Initial seed value for ω (dN/dS) used in the maximum likelihood simulation

[c] Model of codon frequency

[d] Twice the difference in the natural logs of the likelihoods ($\Delta\ell$ x 2) of the two models being compared. This value is used in a likelihood ratio test along with the degrees of freedom. In all cases (M1a-M2a), (M7-M8), (M8a-M8), a model that allows positive selection is compared to a null model. The p-value indicates the confidence with which the null model can be rejected.

[e] The tree length is the number of substitutions per site along all branches in the phylogeny. It is calculated as the sum of the branch lengths, and is a representation of total diversity in the dataset

[f] dN/dS value of the class of codons evolving under positive selection in M8, and the percent of codons falling in that class.

[g] Amino acid positions identified in the class of codons evolving under positive selection in M8 with a posterior probability >0.90. Coordinates correspond to the human protein.
