# Supplementary material for: Ancient and Recent Adaptive Evolution of Primate Non-Homologous End Joining Genes
Source: PLoS Genet. 2010 Oct 21;6(10):e1001169. doi: 10.1371/journal.pgen.1001169 (PMC2958818; doi:10.1371/journal.pgen.1001169)
Supplement: Table S9 — PAML analysis of primate XLF sequences. (0.03 MB PDF) [file pgen.1001169.s010.pdf]

Table S9. PAML analysis of primate XLF sequences.

XLF [a] dataset	ω_0 [b]	codon freq. [c]	M1a-M2a		M7-M8		M8a-M8		tree length [e]	dN/dS (%) [f]	AA Positions of dN/dS>1 [g] * p>0.95 ** p>0.99
			$2\Delta\ell$ [d]	p-value	$2\Delta\ell$ [d]	p-value	$2\Delta\ell$ [d]	p-value			
All 20 primates	0.4	f61	0.30	p=0.863	0.30	p=0.860	0.29	p=0.589	0.68	ns	ns
	0.4	f3x4	0.02	p=0.991	0.07	p=0.967	0.04	p=0.850	0.67	ns	ns
	1.6	f61	0.30	p=0.863	0.30	p=0.860	0.29	p=0.589	0.68	ns	ns
	1.6	f3x4	0.02	p=0.991	0.07	p=0.967	0.04	p=0.850	0.67	ns	ns

[a] Dataset consisted of the aligned primate sequences *Homo sapiens, Pan troglodytes, Gorilla gorilla, Pongo pygmaeus* (Sumatran Orangutan)*, Pongo pygmaeus* (Borneo Orangutan)*, Hylobates syndactylus, Hylobates leucogenys, Hylobates agilis, Macaca mulatta, Macaca fascicularis, Lophocebus albigena, Papio anubis, Miopithecus talapoin, Cercopithecus wolfi, Colobus guereza, Trachypithecus francoisi, Saimiri sciureus, Callithrix jacchus, Callicebus cupreus,* and *Alouatta sara.*

[b] Initial seed value for ω (dN/dS) used in the maximum likelihood simulation

[c] Model of codon frequency

[d] Twice the difference in the natural logs of the likelihoods ($\Delta\ell$ x 2) of the two models being compared. This value is used in a likelihood ratio test along with the degrees of freedom. In all cases (M1a-M2a), (M7-M8), (M8a-M8), a model that allows positive selection is compared to a null model. The p-value indicates the confidence with which the null model can be rejected.

[e] The tree length is the number of substitutions per site along all branches in the phylogeny. It is calculated as the sum of the branch lengths, and is a representation of total diversity in the dataset

[f] dN/dS value of the class of codons evolving under positive selection in M8, and the percent of codons falling in that class.

[g] Amino acid positions identified in the class of codons evolving under positive selection in M8 with a posterior probability >0.90. Coordinates correspond to the human protein.
