# Supplementary material for: Ancient and Recent Adaptive Evolution of Primate Non-Homologous End Joining Genes
Source: PLoS Genet. 2010 Oct 21;6(10):e1001169. doi: 10.1371/journal.pgen.1001169 (PMC2958818; doi:10.1371/journal.pgen.1001169)
Supplement: Table S12 — Summary of human population genetic tests performed on HapMap data. (0.04 MB PDF) [file pgen.1001169.s013.pdf]

Table S12: Summary of human population genetic tests performed on HapMap data

| | | CMS | | IHS | | XPEHH | | FST | |
|---|---|---|---|---|---|---|---|---|---|
| Pop | Gene | P-value | Max Score | P-value | Max Score | P-value | Max Score | P-value | Max Score |
| CEU | POLL | 1.000 | 1.472 | 0.430 | 2.450 | 0.886 | 0.612 | 0.068 | 0.505 |
| CEU | XRCC4 | **0.002** | 4.025 | **0.012** | 3.069 | 0.066 | 2.842 | **0.026** | 0.582 |
| CEU | ARTEMIS | 1.000 | 2.926 | **0.033** | 3.862 | 0.073 | 2.786 | 0.111 | 0.465 |
| CEU | NBS1 | 1.000 | 2.818 | 0.164 | 2.481 | 0.145 | 2.377 | 0.090 | 0.483 |
| CEU | CtIP | 1.000 | 1.731 | 0.252 | 2.329 | 0.118 | 2.506 | 0.341 | 0.361 |
| JPTCHB | POLL | 1.000 | 2.093 | 0.387 | 2.952 | 0.367 | 1.631 | 0.417 | 0.375 |
| JPTCHB | XRCC4 | 1.000 | 2.914 | 0.063 | 3.238 | 0.380 | 1.600 | 0.209 | 0.457 |
| JPTCHB | ARTEMIS | 1.000 | 1.402 | 0.259 | 2.171 | 0.341 | 1.695 | 0.407 | 0.379 |
| JPTCHB | NBS1 | 1.000 | 1.947 | 0.476 | 2.532 | 0.730 | 0.904 | 0.058 | 0.573 |
| JPTCHB | CtIP | 1.000 | 1.383 | 0.501 | 1.828 | 0.466 | 1.424 | 0.587 | 0.322 |
| YRI | POLL | 0.082 | 3.949 | 0.087 | 2.958 | **0.031** | 2.982 | 0.515 | 0.444 |
| YRI | XRCC4 | 1.000 | 2.042 | **0.009** | 3.456 | 0.472 | 1.895 | 0.372 | 0.499 |
| YRI | ARTEMIS | 0.100 | 3.318 | 0.054 | 3.097 | 0.121 | 2.560 | 0.057 | 0.697 |
| YRI | NBS1 | 1.000 | 2.788 | 0.064 | 3.668 | 0.309 | 2.155 | 0.087 | 0.664 |
| YRI | CtIP | 1.000 | 2.759 | 0.142 | 2.551 | 0.497 | 1.856 | 0.206 | 0.578 |
